# A review of the Larainae of Australia with description of seven new species and the new genus *Australara* (Coleoptera, Byrrhoidea, Elmidae)

**DOI:** 10.3897/zookeys.1073.71843

**Published:** 2021-11-29

**Authors:** Cheryl B. Barr, William D. Shepard

**Affiliations:** 1 Essig Museum of Entomology, 1101 Life Sciences Bldg. #4780, University of California, Berkeley, CA 94720 USA University of California Berkeley United States of America

**Keywords:** aquatic beetles, *Hydora*, *Ovolara*, *Potamophilinus*, riffle beetles, *Stetholus*, new taxa, taxonomy

## Abstract

The three genera and four species of Larainae (Elmidae) previously described from Australia are reviewed, and one new genus and seven new species are described: *Australaraglaisteri***gen. et sp. nov.**, *Ovolaralawrencei***sp. nov.**, *Ovolaramonteithi***sp. nov.**, *Stetholuscarinatus***sp. nov.**, *Stetholuslongipennis***sp. nov.**, *Stetholusmetatibialis***sp. nov.**, and *Stetholusworonora***sp. nov.** A lectotype is designated for *Hydoralaticeps* (Carter & Zeck), and the first new collection records of the species are reported since its description in 1932. The occurrence in Australia of *Potamophilinuspapuanus* Satô, described from Papua New Guinea, is reported. A key to the species, photographic images of the external morphology and male genitalia, distribution maps, and habitat and behavioral information, when known, are provided for all twelve species of Australian Larainae.

## Introduction

The aquatic beetle family Elmidae is traditionally divided into two subfamilies, the Elminae and the Larainae. The Elminae are by far the most diverse of the two, with 123 genera and nearly 1,350 recognized species worldwide, whereas the Larainae have only 28 genera with nearly 160 species (Kodada et al. 2016). The laraines are poorly represented in Australia, with just three genera and four species described: *Hydoralaticeps* (Carter & Zeck, 1932), *Ovolaraaustralis* (King, 1865), *Ovolaraleai* (Carter, 1926), and *Stetholuselongatus* Carter & Zeck, 1929. In the 90+ years since being described, little has been published on these taxa or the Australian Larainae in general. Larval rearing studies by Glaister (1985, 1992, 1999) resulted in the association of elmid adults and larvae, enabling her to publish an extensively illustrated identification guide to the larval Elmidae of Australia with keys and descriptive notes on taxonomy, distribution, and habitat (Glaister 1999). Calder (1992), for a Coleoptera identification workshop, produced an unpublished, illustrated adult key to the genera of Australian Elmidae, with taxonomic notes and species-level genitalic illustrations which included all of the described laraine species. Calder (1992) and Glaister (1999) first reported the presence of a fourth laraine genus, *Potamophilinus* Grouvelle, 1896, in Australia.

During a trip to north Queensland in 2001, we collected specimens of an undescribed genus and species and two undescribed species in the genera *Ovolara* and *Stetholus*. Specimens of a *Potamophilinus* species were also collected, which allowed us to identify and confirm the occurrence of *P.papuanus* Satô in Australia. Specimens of a second undescribed species of *Stetholus* were collected in New South Wales in 2019 by European colleagues Martin Fikáček, Matthias Seidel and Vít Sýkora, who provided them for this study. We and other collectors who have searched for enigmatic *Hydoralaticeps*, known only from the type series collected more than 90 years ago, have failed to find additional specimens. However, during our recent examination of material on loan from museum collections, we discovered four previously unidentified specimens of *H.laticeps* in addition to three more new species of *Ovolara* and *Stetholus*. In this article, we describe these seven new species and one new genus, and review the subfamily Larainae of Australia which now includes 12 species in four genera.

## Materials and methods

### Institutional abbreviations

**>AM**The Australian Museum, Sydney, New South Wales, Australia

**ANIC**Australian National Insect Collection, CSIRO, Canberra, Australian Capital Territory, Australia

**EMEC** Essig Museum of Entomology, University of California, Berkeley, California, USA

**MAGNT**Museum and Art Gallery of the Northern Territory, Darwin, Australia

**NMPC** National Museum, Prague, Czech Republic

**NMV**Museums Victoria, Melbourne, Victoria, Australia

**QM**Queensland Museum, South Brisbane, Queensland, Australia

**SAMA**South Australian Museum, Adelaide, South Australia, Australia

**TMAG**Tasmanian Museum and Art Gallery, Hobart, Tasmania, Australia

Geographic abbreviations used in the text include: **ACT** = Australian Capital Territory; **NSW** = New South Wales; **QLD** = Queensland; **NQLD**, **NQ**, **N.Qld**. = north Queensland; **VIC** = Victoria.

### Study material

The authors examined a total of 540 specimens during this project. These were borrowed from Australian institutional collections (>AM, ANIC, QM, SAMA) or were collected by the authors and, in the case of one new species, by European colleagues.

### Field techniques

Specimens collected by the authors were manually dislodged from surfaces and objects, then captured in aquatic nets, or were swept from streamside and emergent vegetation. The collections were placed in vials containing 95 % ethanol in the field and examined later in the laboratory. Related taxa collected with the laraines are reported in the species treatments as “Associated byrrhoid taxa.”

### Laboratory procedures

Examination and measurement of specimens were done with a Leica MZ 12.5, fitted with an ocular micrometer, and an AO Spencer Model 25 stereo microscope. A series of species from each of the authors’ collection localities was dried and point-mounted after genitalic dissection. Specimens on loan also were often dissected for genitalic examination, and those previously glued to card mounts were remounted as was necessary. After study, the genitalia were placed in vials, each containing a drop of glycerin, and affixed to pins below the specimens. For almost all species with sufficient numbers, some specimens were further dissected to view other structures more accurately such as antennae, mouthparts, elytra, and metathoracic wings. The dissected parts were then slide mounted and examined. Measurements of body length consist of the pronotal length plus the elytral length taken at the midline, and do not include the head or the variable space between the pronotum and elytra; measurements of width are of both elytra at their widest point.

### Specimen imaging and distribution mapping

Most of the habitus images were taken using a Visionary Digital BK Plus Lab System fitted with a Canon EOS 7D camera. Some of the images were provided by staff at museums where the specimens are housed, as noted in the figure legends and the Acknowledgments. The genitalia images were taken with a Syncroscopy AutoMontage ® system. Images were prepared and assembled using Adobe Photoshop Elements.

**Figures 1–12.**
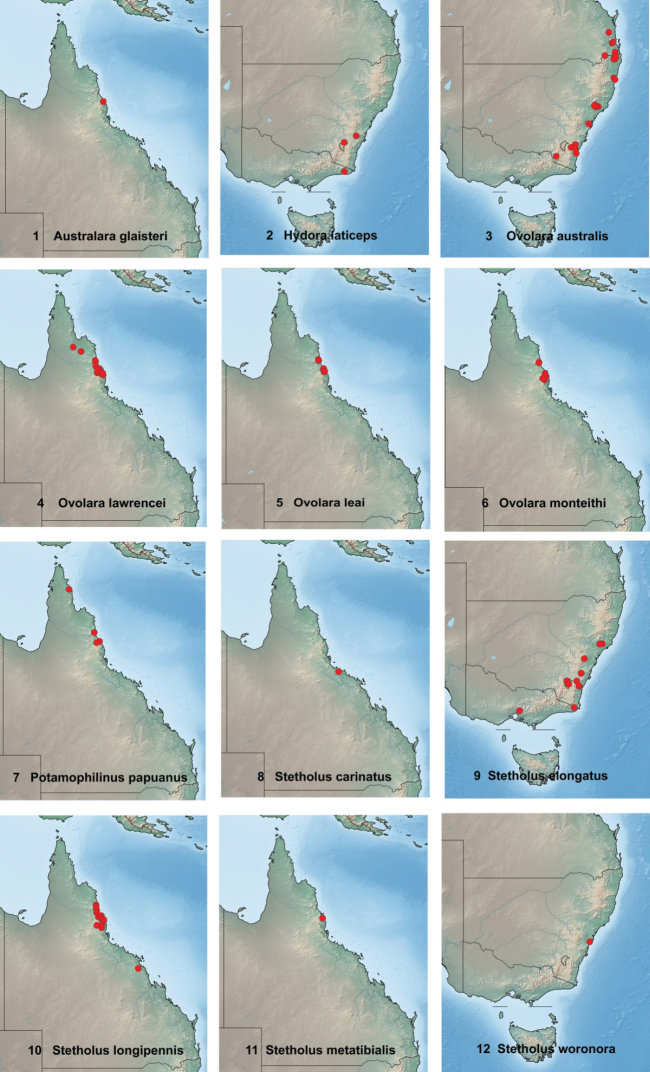
Geographical distribution of species records **1***Australaraglaisteri* gen. nov., sp. nov. **2***Hydoralaticeps***3***Ovolaraaustralis***4***Ovolaralawrencei* sp. nov. **5***Ovolaraleai***6***Ovolaramonteithi* sp. nov. **7***Potamophilinuspapuanus***8***Stetholuscarinatus* sp. nov. **9***Stetholuselongatus***10***Stetholuslongipennis* sp. nov. **11***Stetholusmetatibialis* sp. nov.**12***Stetholusworonora* sp. nov.

SimpleMappr, a free internet program (Shorthouse 2010), was used to create the species distribution maps (Figs 1–12). Geographical coordinates for specimens collected by the authors were obtained using a hand-held GPS unit. For museum specimen locality data, Google Earth Pro was used to acquire geographical coordinates for low-resolution mapping in the SimpleMappr format. This data was obtained from Google Earth maps containing the following attribution: “©2021 Google, Data SIO, NOAA, U.S. Navy, NGA, GEBCO; Data LDEO-Columbia, NSF, NOAA; Image Landsat / Copernicus.”

### Label data

Label data are reported verbatim in the Material Examined, but only the data of primary types (holotypes, lectotype) are enclosed within quotation marks. A single slash “/” indicates the end of a line of text, and a double slash “//” indicates the end of a label. Clarifications, corrections, or missing data may be provided within brackets “ [].” An abbreviation found in the specimen data, besides those of depositories, is WDS-A-[#] = William D. Shepard aquatic field collection number.

## Taxonomy

### Species checklist and distribution of Australian Larainae

*Australaraglaisteri* sp. nov.: NQLD

*Hydoralaticeps* (Carter & Zeck, 1932): ACT, NSW, VIC

*Ovolaraaustralis* (King, 1865): NSW, QLD

*Ovolaralawrencei* sp. nov.: NQLD

*Ovolaraleai* (Carter, 1926): NQLD

*Ovolaramonteithi* sp. nov.: NQLD

*Potamophilinuspapuanus* Sâto, 1973: NQLD

*Stetholuscarinatus* sp. nov.: NQLD

*Stetholuselongatus* Carter & Zeck, 1929: ACT, NSW, VIC

*Stetholuslongipennis* sp. nov.: NQLD

*Stetholusmetatibialis* sp. nov.: NQLD

*Stetholusworonora* sp. nov.: NSW

### Key to the species of Australian Larainae

**Table:** 

1	Pronotum without a distinct transverse impression or impressions anterior to the middle	**2**
–	Pronotum with a distinct transverse impression or impressions anterior to the middle	**7**
2	Body elongate; prosternum not produced anteriorly to form a chin piece; apices of hind tibiae extending beyond elytral apices	**6**
–	Body oval or elliptical; prosternum produced anteriorly to form a chin piece; apices of hind tibiae not extending beyond elytral apices ... ***Ovolara* Brown**	**3**
3	Antennomeres 3–11 forming a stout, ovoid club; pronotum sculptured, midline with a shallow longitudinal sulcus at anterior 2/3 and a broad costa at posterior 1/3 (Fig. 23)	***Ovolaraaustralis* (King)**
–	Antennomeres 3–11 forming an elongate club; pronotum without a prominent longitudinal sulcus or costa	**4**
4	Elytron without an accessory basal stria between striae 1 and 2; male genitalia with penis tapered and narrow, parameres clasping tip at apical 1/3 (Fig. 26); pronotal basal margin protuberant between the prescutellar foveae (Fig. 25)	***Ovolaralawrencei* sp. nov.**
–	Elytron with a very short accessory basal stria of 1–3 punctures between striae 1 and 2, rarely obscure; male genitalia not as above; pronotal basal margin not or only weakly protuberant between prescutellar foveae	**5**
5	Male genitalia with penis narrower at apex than at midlength, parameres not clasping tip (Fig. 31); apical elytral punctures large and deep, similar to those more basal (Fig. 30)	***Ovolaramonteithi* sp. nov.**
–	Male genitalia with penis wider at apex than at midlength, parameres clasping tip (Fig. 29); apical elytral punctures smaller and shallower than those more basal (Fig. 28)	***Ovolaraleai* (Carter)**
6	Pronotum with basal, sublateral carinae; mesoventrite with a moderately wide, deep mesoventral cavity (Figs 15, 20)	***Hydoralaticeps* Carter & Zeck (in part)**
–	Pronotum without basal, sublateral carinae; mesoventrite with a slit-like mesoventral cavity contained within a narrow, anterior projection (Fig. 13)	***Australaraglaisteri* sp. nov.**
7	Elytra with angulate apices; pronotum mostly flat; pronotal posterior angles explanate, each with a distinct oval depression (Fig. 32)	***Potamophilinuspapuanus* Satô**
–	Elytra with rounded apices; pronotum convex; pronotal posterior angles not explanate, at most moderately depressed	**8**
8	Eyes hemispherical, very protuberant; maxillary palpi narrow at apices; prosternum moderately long anterior to coxae (Fig. 22)	***Hydoralaticeps* Carter & Zeck (in part)**
–	Eyes ovoid, not very protuberant; maxillary palpi wide and oblique at apices; prosternum very short anterior to coxae ... ***Stetholus* Carter & Zeck**	**9**
9	Pronotum without basal sublateral carinae	**10**
–	Pronotum with basal sublateral carinae	**11**
10	Antennae distinctly clavate; pronotum moderately sculptured (Fig. 36); male genitalia heavily sclerotized, penis slightly longer than parameres (Fig. 37)	***Stetholuselongatus* Carter & Zeck**
–	Antennae slender, almost moniliform; pronotum lightly sculptured (Fig. 38); male genitalia moderately sclerotized, with penis at least 2 × longer than parameres (Fig. 39)	***Stetholuslongipennis* sp. nov.**
11	Length (excluding head) 5.0 mm or longer; pronotal sublateral carinae very short (Fig. 41)	***Stetholusworonora* sp. nov.**
–	Length (excluding head) 4.0 mm or shorter; pronotal sublateral carinae 1/3–1/2 as long as pronotum	**12**
12	Metatibiae with posterior surfaces glabrous and shiny; elytral accessory basal stria between striae 1 and 2 with several distinct punctures (Fig. 40)	***Stetholusmetatibialis* sp. nov.**
–	Metatibiae entirely setose; elytral accessory stria with only a few faint punctures (Fig. 34)	***Stetholuscarinatus* sp. nov.**

#### Australara gen. nov.

Taxon classificationAnimaliaColeopteraElmidae

03DD3AC4-A626-5764-99ED-2916C7DCB625

http://zoobank.org/47BFA6C2-8BDE-4CFB-B7A7-C4299E45CAFB

##### Type species.

*Australaraglaisteri* sp. nov.

##### Differential diagnosis.

**Figures 13, 14.**
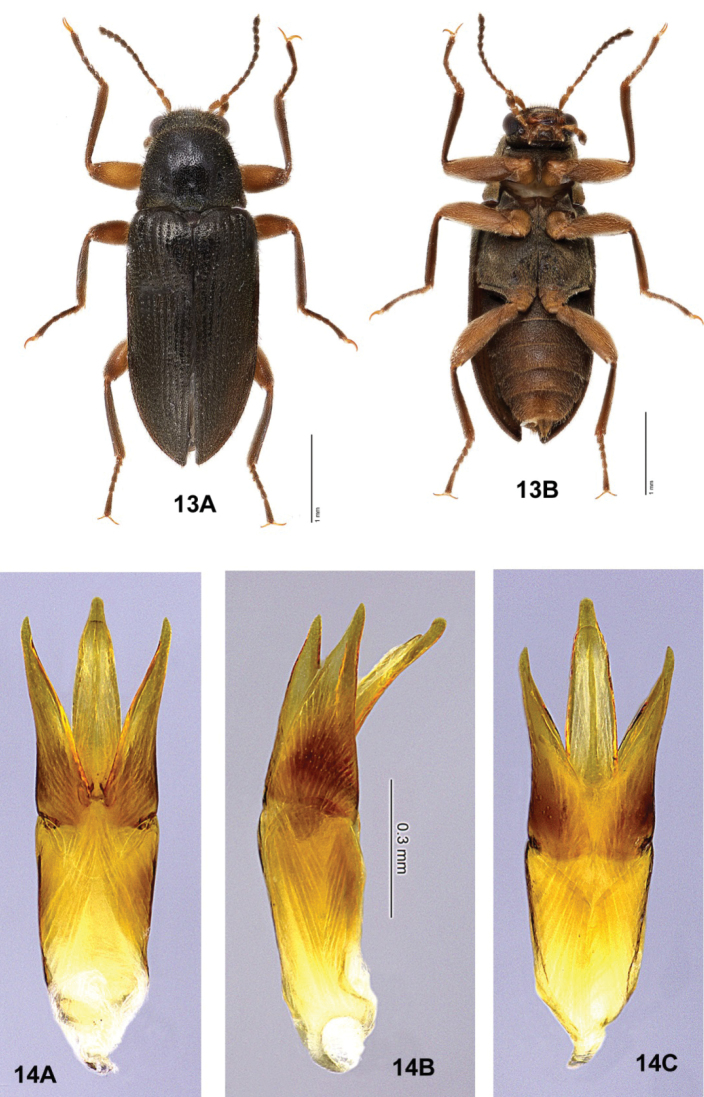
*Australaraglaisteri* gen. nov., sp. nov., male **13** habitus **A** dorsal **B** ventral (photographs courtesy of the Australian National Insect Collection, CSIRO, Zhenhua Liu) **14** male genitalia **A** dorsal view **B** lateral view **C** ventral view.

*Australara* (Fig. 13) is distinguished by the following characters: Body shape elongate; antennae subserrate, thin, very long; eyes moderately protuberant; maxillary palpi long and robust, apices tapered, sensory areas oblique and narrowly oval; labial palpomere 3 apex with white, digitiform, sensory area; pronotum with two faint, anterior transverse impressions laterad of the midline, basal sublateral carinae absent; prosternum moderately short anterior to procoxae, not extending beneath head; prosternal process spinose; mesoventrite with a narrow projection from the anterior margin containing a slit-like mesoventral cavity to receive the prosternal process; apices of hind tibiae exceeding the elytral apex.

**Figure 15.**
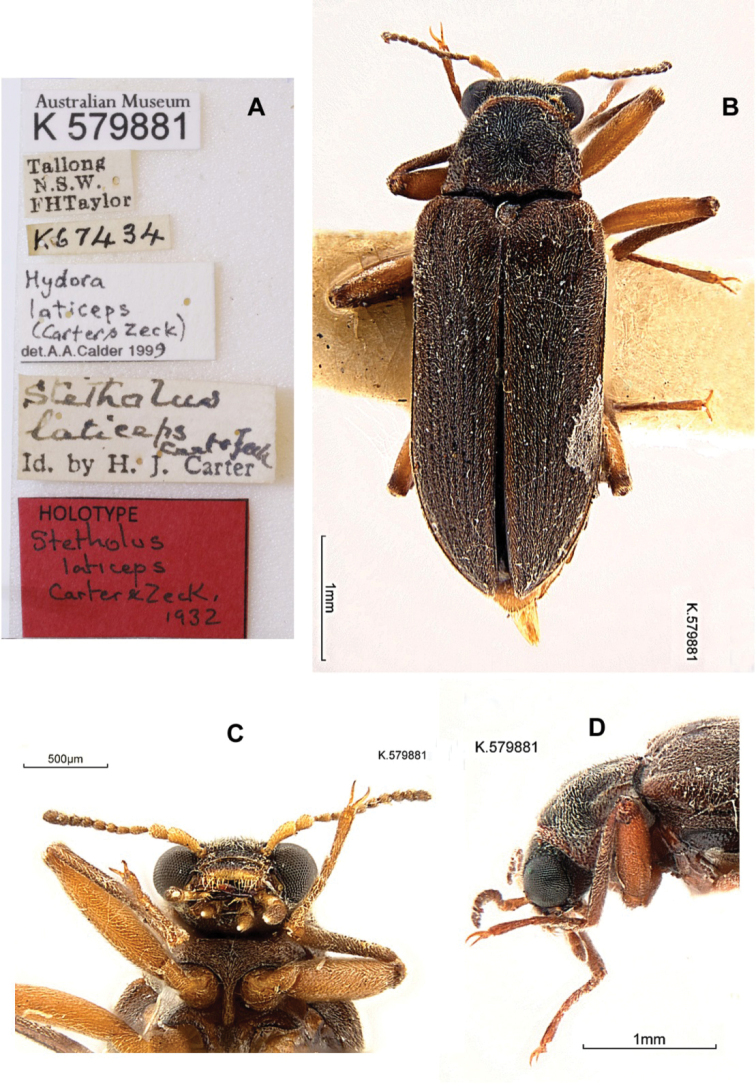
*Hydoralaticeps*, lectotype male **A** specimen labels **B** dorsal habitus **C** ventral head and prosternum **D** lateral head and pronotum (photographs courtesy of the Australian Museum, Natalie Tees).

*Stetholus* species (Figs 34–42), with similarly elongate bodies, are differentiated by the antennae (shorter, distinctly clavate), maxillary palpi (apical sensory area strongly oblique to base of palpomere 4 and widely open); prosternum (very short anterior to the procoxae), mesoventrite (mesoventral cavity large and deep, not within an anterior projection), and length of the hind legs (tibiae not exceeding elytral apices). *Hydora* (Figs 15B–D, 17–20, 22), like *Australara*, has an elongate body, long hind legs, and similar maxillary palpi, but differences include characteristics of the eyes (hemispherical, very protuberant), pronotum (with basal sublateral carinae), and mesoventrite (mesoventral cavity deep and moderately wide, not within an anterior projection).

##### Description.

Body elongate; setose, setae longer and more dense on venter than on dorsum. Antenna thin, very long, antennomeres 3–11 subserrate; eye moderately protuberant, subcircular at base; maxillary palpus long and robust, palpomere 4 with apex tapered, sensory area oblique, narrowly oval. Pronotum lightly sculptured with a pair of faint anterior transverse impressions and a pair of elongate basal sublateral impressions. Elytron marginate, shallowly punctate and striate, apex acute. Prosternum moderately short anterior to procoxae, not extending anteriorly beneath head; prosternal process long, spinose, carinate. Mesoventrite with a narrow projection from the anterior margin containing a slit-like mesoventral cavity. Abdominal ventrites 1 and 2 combined much shorter than ventrites 3–5 combined. Legs long, slender, apex of hind tibia extending beyond elytral apex.

##### Etymology.

From the Latin *australis*, meaning southern, in reference to the Southern Hemisphere as well as the continent of Australia, plus *Lara*, the type genus of the subfamily Larainae.

##### Distribution.

Known only from the type locality in north Queensland, Australia (Fig. 1).

##### Comments.

Described from only three specimens, all males, from one locality. The larva is unknown.

#### Australara glaisteri sp. nov.

Taxon classificationAnimaliaColeopteraElmidae

CF235683-DA28-5A49-98C3-4DEC536A0F7A

http://zoobank.org/BA2ACE21-58F3-41FD-92A8-7107B10055D9

Figs 1 , 13 , 14

##### Type locality.

Mulgrave River south of Gordonvale; 17.1028°S, 145.7875°E; north Queensland, Australia.

##### Type material.

***Holotype*, male.** “AUSTRALIA: Queensland / 1 km S Gordonvale / 18 I 2001 94 ft / Mulgrave River (WDS-A-1371 on reverse) // William D. / Shepard, leg. // HOLOTYPE / Australara / glaisteri / Barr & Shepard” [red label, handwritten]. Dry pinned. Deposited in the Australian National Insect Collection, Canberra; ANIC Database Number 25-077640**. *Paratypes*** (2). Same data as for holotype // PARATYPE / *Australara* / *glaisteri* / Barr & Shepard [yellow label, printed] (2 ♂♂ EMEC).

##### Differential diagnosis.

*Australaraglaisteri* (Figs 13, 14) may be separated from other laraine species by the characters given in the generic diagnosis: *Hydoralaticeps* (Figs 15–22) shares some similarities, but differs by having strong sublateral pronotal carinae, whereas *A.glaisteri* has none. Unlike *A.glaisteri*, *Stetholus* species (Figs 34–42) have shorter, distinctly clavate antennae, and the prosternum is very short anterior to the procoxae. The male genitalia of *A.glaisteri* (Fig. 14) are unusual, with the penis curved strongly in a dorsal direction.

##### Description

**(n = 3). Male. *Body***: Size 3.7–4.0 mm long, 1.4–1.5 mm wide; elongate, 2.5–3 × longer than wide. Dorsal color very dark brown; venter medium brown; head black; first 2 antennomeres, basal palpomeres, coxae, trochanters, femora yellow-brown; tibiae, tarsi, apical antennomeres, apical palpomeres brown. All surfaces with short to moderately long yellow setae, setae more dense ventrally than dorsally; dorsal cuticle shiny. ***Head***: Densely and finely punctate, punctures 1 diameter apart or less; moderately setose, setae moderately long. Frons moderately protuberant between eyes, with adjacent lateral excavations and a pair of fossae above antennal bases; frontoclypeal suture straight. Antenna with eleven antennomeres, very long, thin, forming a loose, slightly asymmetrical club; antennomere 1 longest, ~ 3 × longer than wide, slightly curved; antennomere 2 ovoid; antennomeres 3–10 subserrate, with antennomeres 5–10 subequal in size; antennomere 11 broadly ovoid. Eye finely faceted, almost circular at base, moderately protuberant; dorsal margin with fringe of long, curved setae. Clypeus transverse, very short, ~ 7 × wider than long; anterior margin weakly emarginate; disc granulate; lateral margins with long setae. White membranous area visible between clypeus and labrum. Labrum rectangular, > 2 × wider than long, longer and wider than clypeus; anterior margin straight; disc granulate, very setose; lateral margins broadly rounded with long, yellow setae. Mandible with three teeth, apical pointed, 2^nd^ triangular, 3^rd^ smallest and triangular; lateral margins with several long setae. Maxillary palpus long, robust, setose, with four palpomeres; palpomere 1 short, annular; palpomere 2 twice as long as wide; palpomere 3 shorter and wider than 2, wider apically; palpomere 4 wide, ovoid, apex angled obliquely, ventral surface with a narrowly oval, white sensory area. Galea and lacinia long, finger-like, both with long setae. Labial palpus long, robust, yellow, with three setose palpomeres; palpomere 1 short and narrow, annular; palpomere 2 twice as wide as 1; palpomere 3 conical, apex with white, digitiform, sensory area. ***Pronotum***: Shape nearly quadrate, slightly wider than long, widest at base; 0.9–1.0 mm long, 1.1–1.2 mm wide; disc densely punctate, punctures spaced ~ 1 diameter apart. Anterior margin thickened, straight; anterior angles obsolete; lateral margins weakly sinuate, marginate; posterior angles depressed, lateral margins raised, variably produced with tips generally blunt; posterior margin weakly trisinuate. Disc moderately convex; two faint, anterior transverse impressions laterad of the midline at anterior 1/4; two faint to distinct shallow, elongate, sublateral impressions ~ 1/3 length of pronotum; two prescutellar foveae joined by a shallow, transverse impression. ***Scutellar shield***: As long as wide, apex rounded; flat; densely setose. ***Elytron***: 2.8–3.0 mm long, 0.7–0.8 mm wide. Elytra conjointly 2 × as long as wide; generally parallel-sided; laterally compressed at basal 1/2; lateral margins strongly marginate. Humerus inflated, elytral base slightly depressed; disc moderately convex at anterior 1/4 median to humerus; moderately depressed at anterior 1/4–1/3 posterior to humerus; then weakly convex to apex. Disc with ten small, shallowly punctate, weakly impressed striae, intervals nearly flat; short, faint, accessory basal stria with close to ten punctures between striae 1 and 2; striae 2 and 3 end before apex; disc punctures of variable size, separated by < 1 diameter, more distinct basally, smaller and closer apically. ***Metathoracic wings***: Macropterous. ***Prosternum***: Moderately short anterior to procoxae, disc very setose with widely spaced punctures; prosternal process spinose, long, 5 × longer than wide, carinate with carina extending anterior of procoxae, apex narrowly rounded. ***Mesoventrite***: Very setose; surface elevated at midline anterior to mesocoxae to form a narrow projection from the anterior margin with two carinae enclosing a slit-like mesoventral cavity; area anterior to mesocoxae shallowly excavated for procoxae; disc depressed between mesocoxae; posterior margin emarginated medially. ***Metaventrite***: Broadly rectangular; very setose, moderately granulate; anterior margin moderately produced between mesocoxae; disc laterally convex, medially with a shallow, wide concave area surrounding discrimen; discrimen extending from anterior 1/4 to posterior margin, deeply incised; metakatepisternal suture distinct. ***Legs***: Long, slender, of similar lengths; each leg with femur and tibia subequal in length; tarsus with tarsomere 5 distinctly shorter than tarsomeres 1–4 combined. Coxae and femora yellow-brown; tibiae brown, each with a pair of stout spines at ventral apex; meso- and metatibiae with posterior surfaces shallowly sulcate, yellow-brown, glabrous, shiny; tarsi brown; claws simple, long, sharply acute. ***Abdomen***: Strongly convex, lateral margins concealed by elytra; densely setose and moderately granulate; with five ventrites, ventrites 1–4 of subequal length, ventrite 5 slightly longer; ventrite 1 with a long, narrow median, triangular intercoxal projection; ventrite 5 posterior margin with a median emargination. ***Aedeagus***: Approximately 3.5 × longer than wide, generally parallel-sided at basal 3/4; phallobase longer than parameres, penis slightly longer than parameres (Fig. 14). Parameres, in dorsal view (Fig. 14A), widest at base; lateral margins nearly parallel at basal 1/2, then weakly divergent at apical 1/2; medial margin weakly arcuate; apex produced, acute. Penis, in dorsal view, widest basally, lateral margins evenly convergent to rounded apex; no visible corona; basal apophyses short, < 1/4 as long as phallobase, straight, broad, blunt at tips. In lateral view (14B), penis strongly curved dorsally above parameres at ~ 30° angle, apex rounded; paramere triangular, apex produced, acute. Fibula absent.

##### Variation.

The three specimens varied in size from 3.7–4.0 mm long and 1.4–1.5 mm wide. Because the small series of *A.glaisteri* is all male, it was not possible to make a comparison with the female of the species. Among the three, the two shallow, elongate, sublateral pronotal impressions vary from faint to distinct. Also, the posterior pronotal angles differ in the amount to which they are produced, the shape of the angle (nearly 90° to acute), and whether the tip is truncate, blunt, or sharp. It is possible that the median emargination on the posterior margin of abdominal ventrite 5 is a male characteristic not present in females.

##### Etymology.

The specific epithet *glaisteri*, a noun in the genitive case, is given in honor of Alena Glaister of Monash University, VIC, who devised a successful method of rearing Australian larval elmids to adults, thereby enabling their association. She published an extensively illustrated identification guide to the larval Elmidae of Australia with keys and descriptive notes on taxonomy, distribution, and habitat. Few elmid researchers have attempted such work, and none have produced larval keys covering so many taxa.

##### Distribution.

Known only from the type locality in north Queensland, Australia (Fig. 1).

##### Habitat.

At the collection site during low water stage, the Mulgrave River was mostly shallow, with warm, clear water and a fairly swift current over a substrate of sand and gravel. Decomposing wood and log jams, where *Australara* and other laraines were found, were abundant along the banks of the wide channel. The locality is at ~ 30 m elevation and bordered by a town and sugarcane fields not far from the ocean. Local residents told us that in past years saltwater crocodiles frequented the river until the sugarcane farmers shot them out.

##### Associated byrrhoid taxa.

Elmidae: Larainae: *Ovolaraleai*, *O.monteithi* sp. nov., *Stetholuslongipennis* sp. nov.; Elminae: *Austrolimnius* spp., *Graphelmispallidipes* (Carter), *Kingolus* spp., *Notriolus* spp., *Simsonia* spp.

#### Genus Hydora

Taxon classificationAnimaliaColeopteraElmidae

Anon. [Broun], 1882

B24CE0B6-8984-5F97-A5BA-06EB07B1D254

##### Type species.

*Pachycephalapicea* Broun, 1881.

##### Diagnosis.

Body elongate, sides subparallel; antennae loosely clavate; eyes large, prominent; pronotum with two basal sublateral carinae; prosternum not extended anteriorly beneath head; elytra striate-punctate, punctation sometimes reduced (New Zealand species), each elytron with one accessory basal stria between striae 1 and 2, elytral apices narrowly rounded; tarsi each with tarsomere 5 subequal to or shorter than tarsomeres 1–4 combined; abdominal ventrites 1 and 2 combined shorter than ventrites 3–5 combined.

##### Distribution.

The genus *Hydora* has an interesting geographic distribution, with ten recognized species occurring in New Zealand (7), Australia (1), and Argentina and Chile (2) (Spangler and Brown 1981; Lambert et al. 2014). There are many currently undescribed species in New Zealand and one in Chile (R. Leschen, V. Sýkora, in litt.).

##### Habitat and behavior.

There is no information available pertaining to the habitat and behavior of *Hydora* in Australia, except for the fact that at least half of the known specimens were collected at lights. In New Zealand, larvae and adult *Hydora* are common on the bottom substrate, or on vegetation, including bryophytes, at the margins of moderate to fast flowing streams (Lambert et al. 2014). Adults can sometimes be found running around on the emergent parts of boulders and have been observed in mass swarms above the water surface (Lambert et al. 2014).

##### Comments.

In this genus the prosternal process may or may not have a median longitudinal carina, depending on the species. Some New Zealand species do, and some do not (Broun 1914; Lambert et al. 2014). No carinae were mentioned in the descriptions of the prosternal processes of the two species from Austral South America, *Hydoraannectens* Spangler & Brown and *H.lenta* Spangler & Brown (Spangler and Brown 1981), and upon examining specimens of those species, we found none. The description of *H.laticeps* from Australia stated that the process is without a carina (Carter and Zeck 1932), but this is debatable. The prosternal process is discussed in the Comments section of the species treatment. The larva was keyed and illustrated at the generic level in Glaister (1999) based on New Zealand specimens.

#### Hydora laticeps

Taxon classificationAnimaliaColeopteraElmidae

(Carter & Zeck, 1932)

F6F0B4DF-411C-5A9B-9BA3-B484B07774F1

Figs 2 , 15 , 16 , 17–19 , 20, 21 , 22

##### Type locality.

Upper Shoalhaven River, Tallong; 34.700°S, 150.083°E (approximate); New South Wales, Australia (lectotype deposited in the Australian Museum, Sydney). Note: The geographic coordinates given in the >AM database place the type locality north of Tallong, whereas the Shoalhaven River is to the south.

##### Type material examined

**(2). *Lectotype* male (here designated). New South Wales.** “Australian Museum / K 579881 // Tallong / N.S.W. / FHTaylor // Stetholus / laticeps / Carter & Zeck / Id. by H. J. Carter // K67434 // HOLOTYPE / Stetholus / laticeps / Carter & Zeck, / 1932 [red label] // Hydora / laticeps / (Carter & Zeck) / det. A.Calder 1999 // LECTOTYPE / Stetholuslaticeps / Carter & Zeck, 1932” [red label, handwritten]. Deposited in the Australian Museum, Sydney. ***Paralectotype* male (here designated). New South Wales.** Australian Museum / K 579882 // Tallong / N.S.W. / FHTaylor // K69264 // Stetholus / laticeps / Carter 1932 // PARATYPE / Stetholuslaticeps / Carter & Zeck, / 1932 [blue label] // Hydora / laticeps / (Carter & Zeck) / det. A.Calder 1999 // PARALECTOTYPE / *Stetholuslaticeps* / (Carter & Zeck, 1932) [yellow label, printed]. Deposited in the Australian Museum, Sydney.

##### Type material examined from photographs

**(2). *Paralectotype* males (here designated). New South Wales.** Tallong / N.S.W. / FHTaylor // Stetholus / laticeps C & Z / Id. by H. J. Carter // Hydora / laticeps (C&Z) / det. A.Calder 1992 // Genitalia prep. / HH-224 ♂/ A. Calder 198792 // PARALECTOTYPE / *Stetholuslaticeps* / (Carter & Zeck, 1932) [yellow label, printed] (1 ANIC); Tallong / N.S.W. / FHTaylor // Stetholus / elongatus / C & Z / Id. by H. J. Carter // Paratype [blue label, printed] // PARATYPE [blue label, printed] // Genitalia prep. / HH-247 ♂/ A.Calder 1997 // ANIC / Image // PARALECTOTYPE / *Stetholuslaticeps* / (Carter & Zeck, 1932) [yellow label, printed] (1 ANIC).

##### Other material examined

**(4). Australian Capital Territory.** AUSTRALIA: / Lyneham / at light / A.C.T. 22.xii.66 / B.P.Moore (1♂ 1♀ ANIC). **Victoria.** Cann River, E.Vic. / 28.i.1967. / G. Monteith // EX UQIC / DONATED / 2011 (2♀♀ QM).

##### Differential diagnosis

**Figure 16.**
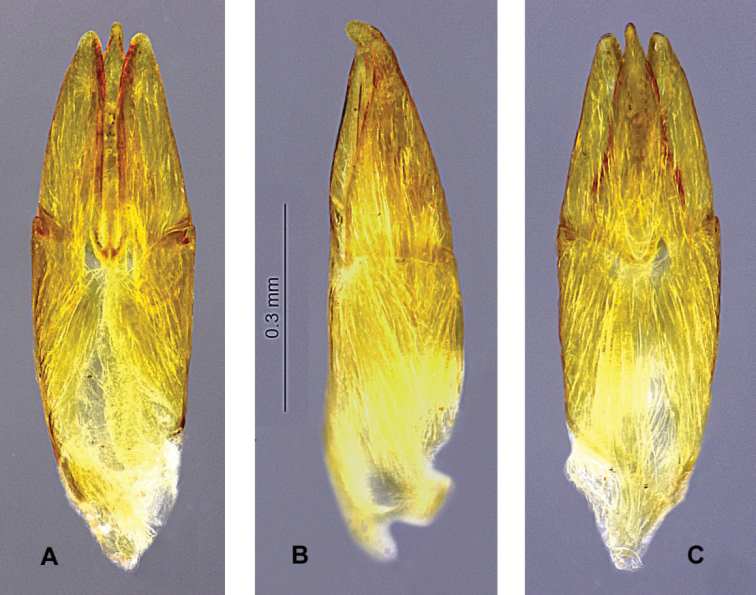
*Hydoralaticeps*, lectotype; male genitalia **A** dorsal view **B** lateral view **C** ventral view.

**Figures 17–19.**
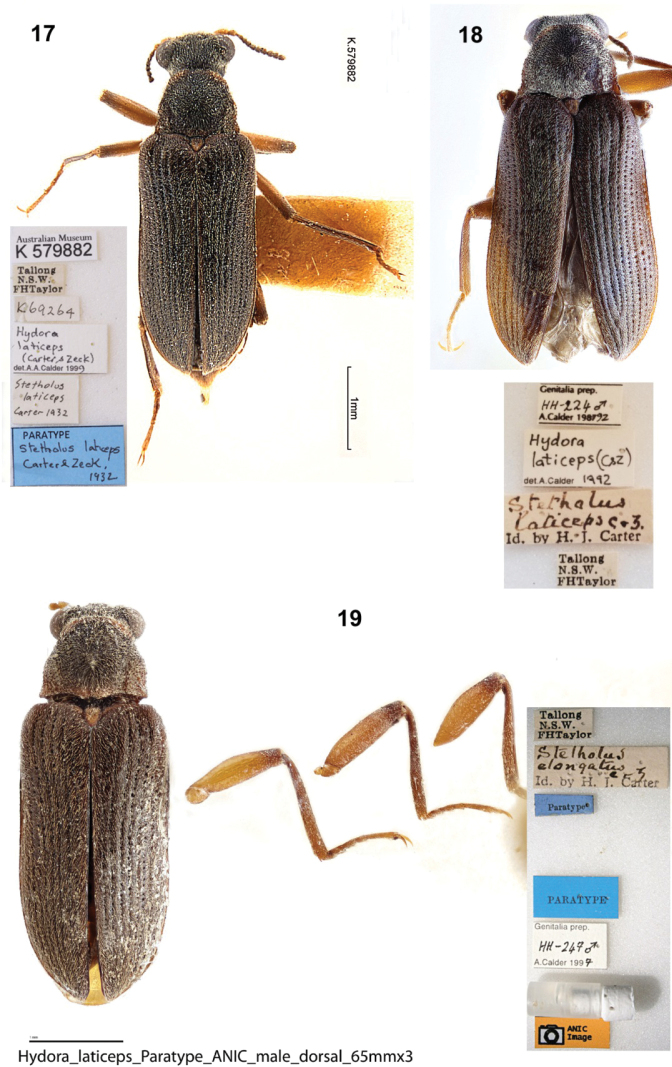
*Hydoralaticeps*, paralectotype males; dorsal habitus with specimen labels **17**>AM specimen (photograph courtesy of the Australian Museum, Natalie Tees) **18**ANIC specimen (photograph courtesy of Vít Sýkora, Charles University, Prague, Czech Republic) **19**ANIC specimen (photograph courtesy of the Australian National Insect Collection, CSIRO).

**Figures 20, 21.**
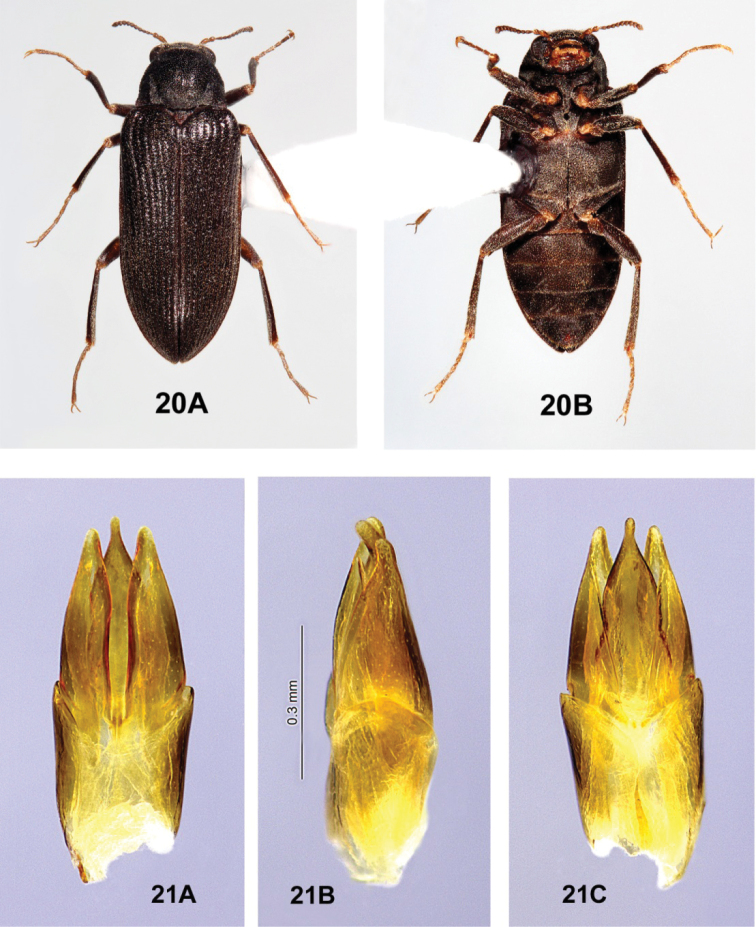
*Hydoralaticeps*, non-types from Lyneham, ACT**20** female habitus, 4.5 mm long **A** dorsal **B** ventral **21** male genitalia **A** dorsal view **B** lateral view **C** ventral view.

**Figure 22.**
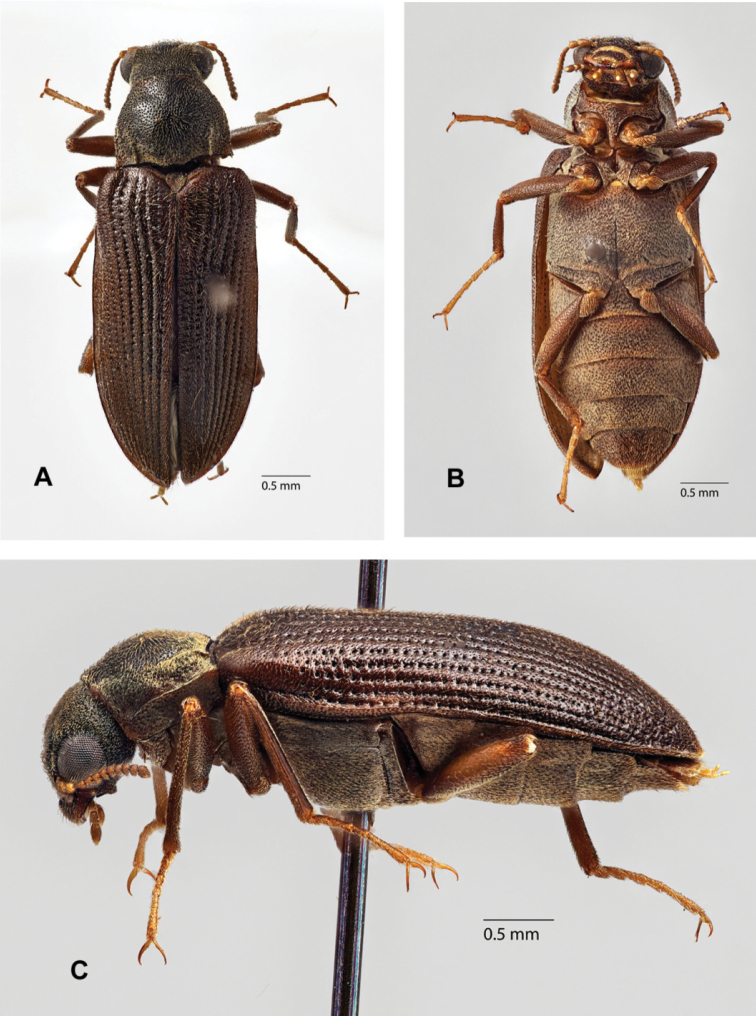
*Hydoralaticeps*, non-type female from Cann River, VIC**A** dorsal habitus **B** ventral habitus **C** lateral habitus (photographs courtesy of the Queensland Museum, Geoff Thompson).

**(n = 8).***Hydoralaticeps* (Figs 15–22) is the only species of *Hydora* known to occur in Australia. It can be distinguished from other Australian laraines by a combination of the following characters: Eyes protuberant, hemispherical; maxillary palpi narrow at the apices; pronotum with strong basal, sublateral carinae and without a distinct transverse impression at anterior 1/3; and prosternum moderately long anterior to the coxae but not extending beneath head. *Stetholus* species (Figs 34–42) have ovoid eyes, not usually prominent; maxillary palpi each with palpomere 4 wide and oblique at the apex; pronotum with a distinct transverse impression; and prosternum very short and narrow anterior to the coxae.

*Australaraglaisteri* (Fig. 13) most obviously differs by its lack of sublateral pronotal carinae and by the mesoventrite having an anterior projection containing a slit-like mesoventral cavity; the eyes are also not quite as protuberant.

##### Redescription

**(n = 2). Male lectotype and male paralectotype. *Body***: Size 4.2 mm long, 1.6 mm wide (lectotype); size 4.0 mm long, 1.5 mm wide (paralectotype); elongate, parallel-sided. Color light to dark brown; head and pronotum darkest; antennae, mouthparts, legs, venter lightest. Dorsum with fine, pale setae, short on elytra, longer on head and pronotum; venter with long, dense setae. ***Head***: Eye large, protuberant, hemispherical. Antenna with antennomere 1 elongate, antennomere 2 ovoid, antennomeres 3–11 smaller, weakly clavate. Labrum emarginate anteriorly, lateral margins with long setae. Maxillary palpus long, robust, setose; palpomere 4 much enlarged, ovoid, apex blunt with small, oval sensory area. Labial palpus shorter, palpomere 4 conical, apex pointed with very small, circular sensory area. ***Pronotum***: Shape generally trapezoidal, 0.9 mm long, 1.1 mm wide (at base); anterior angles obscure, lateral margins crenulate, posterior angles acute, depressed; disc weakly sculptured except for two distinct, basal, sublateral carinae, 1/2 the pronotal length; two shallow, obscure transverse impressions laterad of midline at anterior 1/5–1/4. ***Elytron***: 3.2–3.3 mm long, 0.7–0.8 mm wide (at base); lateral margin narrowly marginate, apex narrowly rounded, acute; disc with ten rows of moderately striate punctures, accessory basal stria present between striae 1 and 2; disc in lateral view flattened at anterior 1/2. ***Prosternum***: Moderately long anterior to coxae, not extending beneath head; prosternal process narrow, curved, posterior 1/3 semi-carinate with a short, faint row of granules at midline, tip narrowly rounded. ***Mesoventrite***: Longer than prosternum; mesoventral cavity deep and moderately wide ***Metaventrite***: Very convex, especially in lateral view. ***Legs***: Long and slender. Tibia of all legs with a pair of stout spines at ventral apex; meso- and metatibia with posterior surfaces shallowly sulcate, glabrous, shiny. Tarsus with tarsomere 5 shorter than tarsomeres 1–4 combined; covered with short, dense setae; claws simple, slender, acute. ***Abdomen***: Ventrite 1 triangular intercoxal projection moderately narrow; ventrite 5 nearly truncate at apex. ***Aedeagus***: Phallobase longer than parameres and penis, penis slightly longer than parameres (Fig. 16). Phallobase open dorsally. In dorsal view (Fig. 16A), parameres broad, with lateral margins gradually convergent, apices bluntly rounded; medial margins parallel-sided at basal 2/3 then gradually divergent, margins appearing more sclerotized than rest of parameres. Penis slightly longer than parameres, approximately as wide at base as paramere base; lateral margins widened and arcuate just distal to base, then evenly convergent to apex; apex narrow, nipple-like, laterally flattened, tip narrowly rounded; no corona visible; basal apophyses short, 1/4–1/3 as long as phallobase, straight, broad, blunt at tips. Fibula absent. In lateral view (Fig. 16B), paramere nearly straight dorsally at apical 3/4, weakly arcuate ventrally, tip broadly rounded and slightly wider than paramere tip.

##### Variation.

There is some size variation among the known specimens, particularly between males and females. Specimens from the type series, all males (n = 4), measured 4.0–4.3 mm long, 1.4–1.6 mm wide. Carter and Zeck (1932) stated in the type description “Dimensions: 5 × 1.5 mm” but the length probably included the head. Among the specimens examined (including two from the type series), the females (n = 3), 4.5–5.2 mm long, 1.7–1.8 mm wide, are considerably larger than the males (n = 3), 4.0–4.5 mm long, 1.4–1.6 mm wide. In addition, the females (Figs 20B, 22B) have prosternal processes broader than those of the males (Fig. 15C), and noticeably narrower maxillary palpi. The prosternal processes of the two male specimens examined from the type series (Fig. 15C) are slightly narrower than those of the non-type male. The surface of the prosternal process varies, and may be convex, depressed only between the procoxae, or entirely flat except posterior to coxae, but in all specimens the process is granulate, swollen, and an indistinct carina is usually visible. Non-sexual variation was also observed in the morphology of the elytral punctures (size and depth), pronotum (width, lateral margins, posterior angles, sculpturing); and prosternal process (width, surface features). On the pronotum, two shallow, anterior, transverse impressions are present laterad of the midline. In most specimens the impressions are weak or altogether obscure (Figs 17–19), but they are quite obvious in one of the two non-type specimens from Cann River (Fig. 22A). In addition, the single male non-type specimen from Lyneham (Fig. 21A, C) has a slightly broader aedeagus than the two specimens examined from the type series (Fig. 16A, C). This variability in external morphology and male genitalia raises the possibility that more than one species is involved.

##### Lectotype designation.

It appears that none of the four known specimens from the type series of *Stetholuslaticeps* was given a holotype or paratype label at the time of description, and those subsequently added to the specimens were not done so by the authors. In their description, Carter and Zeck (1932) stated that they had “five examples” and that the holotype was “in Coll. Carter,” but did not mention designating paratypes. Two specimens were deposited at the Australian Museum 35 years apart: According to the original register of specimens, the first (Fig. 15) was presented by H. J. Carter in 1936, and bears an old, handwritten determination label saying “Stetholuslaticeps Carter & Zeck Id. by H. J. Carter” (Fig. 15A). The specimen was subsequently given a holotype label by an unknown person, i.e., it was not written in the same hand as the determination label by Carter and appears much newer. This specimen is here designated as the lectotype to fix the concept of *Hydoralaticeps* (Carter & Zeck). The second specimen (Fig. 17) was obtained from the late E. H. Zeck in 1971, lacks an original determination label, and likewise bears a newer paratype label; it is designated a paralectotype.

There are also two specimens housed at Australian National Insect Collection. One of them bears the surprising, original determination label “Stetholuselongatus C & Z Id. by H. J. Carter” and two printed paratype labels, one older and one newer (Fig. 19). Probably the identification predated the description of *S.laticeps* by Carter and Zeck in 1932, however, Tallong was not among the localities cited in their 1929 description of *S.elongatus* (Carter and Zeck 1929). The other specimen has an original determination label, “Stetholuslaticeps C & Z Id. by H. J. Carter,” but has no paratype label (Fig. 18). These two specimens are likewise designated as paralectotypes. We were unable to examine the ANIC specimens because they were on loan to another researcher, but we were provided with habitus images (Figs 18, 19) and measurements of body length for this article.

The location of the fifth specimen from the type series is unknown. Lambert et al. (2014) cited the SAMA as a specimen depository for the species, but we have examined their material and found no specimens of *H.laticeps*, so this report was in error.

##### Distribution.

*Hydoralaticeps* is known from only three localities in Australia: the Shoalhaven River near Tallong, New South Wales, the type locality; Lyneham, Australian Capital Territory; and Cann River, eastern Victoria (Fig. 2).

##### Associated byrrhoid taxa.

Elmidae: Larainae: *Stetholuselongatus* (>AM, ANIC, NMV, SAMA); Elminae: *Notriolus* sp. (>AM).

##### Comments.

*Hydoralaticeps* was originally described in the genus *Stetholus* by Carter and Zeck (1932), and subsequently reassigned to *Hydora* by Hinton (1935). Hinton stated that he studied only the description and figures, not actual specimens, and gave no specific reasons for the new combination.

In the diagnosis following their description, Carter and Zeck (1932: 203) noted that the prosternal process of *S.laticeps* lacks a carina, in contrast to *Stetholuselongatus* Carter & Zeck (1929) which has a carina. The actual situation is less clear-cut. The surface of the apical 1/2-1/3 of the process is convex to varying degrees and may be somewhat granulate at the midline, resembling an indistinct carina. However, this is a poor diagnostic character because dense setation can make examination difficult.

When Carter and Zeck (1932) described *S.laticeps* they made no mention that *S.elongatus*, described by them in 1929, was present in the Upper Shoalhaven River as well. That *S.elongatus* was collected with *H.laticeps* at the type locality is evidenced by museum specimens with locality labels identical to those of *H.laticeps*: “Tallong N.S.W. FH Taylor.” This raised the question as to whether the missing specimen of *H.laticeps* might bear a *S.elongatus* label, as does one of the ANIC specimens, and thus has been overlooked. Unfortunately, examination of all known *S.elongatus* specimens with collection labels as above (>AM, 3 specimens; ANIC, 3; NMV, 2; SAMA, 4) revealed no misidentifications.

Until now, *Hydoralaticeps* has been known only from its type locality, the Upper Shoalhaven River near Tallong, New South Wales, Australia. In the 90+ years since the type series was collected, deliberate attempts to re-collect it have been unsuccessful. Examination of unidentified museum specimens for this project resulted in the discovery of four additional specimens from two new localities, all of which were collected at light. The four type specimens available are all males, the Lyneham specimens are male and female, and the Cann River specimens are both female. In the absence of males, the latter two specimens are assumed to be *H.laticeps* due to external morphological similarities. The larva of the species is unknown.

As mentioned in the Variation section, is possible that not all of the specimens are conspecific because of mophological variation which is apparent even among those from the type series. However, there is not enough evidence at present to assign any to a species other than *H.laticeps*. DNA analysis would be helpful in this regard if fresh material could be obtained. A recent attempt to obtain DNA from a specimen in the type series failed due to its age (V. Sýkora, in litt.), and even the youngest of the specimens is at least 54 years old.

#### Genus Ovolara

Taxon classificationAnimaliaColeopteraElmidae

Brown, 1981

49EB0E99-8991-5E00-A96F-698E21D47A9B

##### Type species.

*Lutochrusaustralis* King, 1865.

##### Diagnosis.

**Figures 23, 24.**
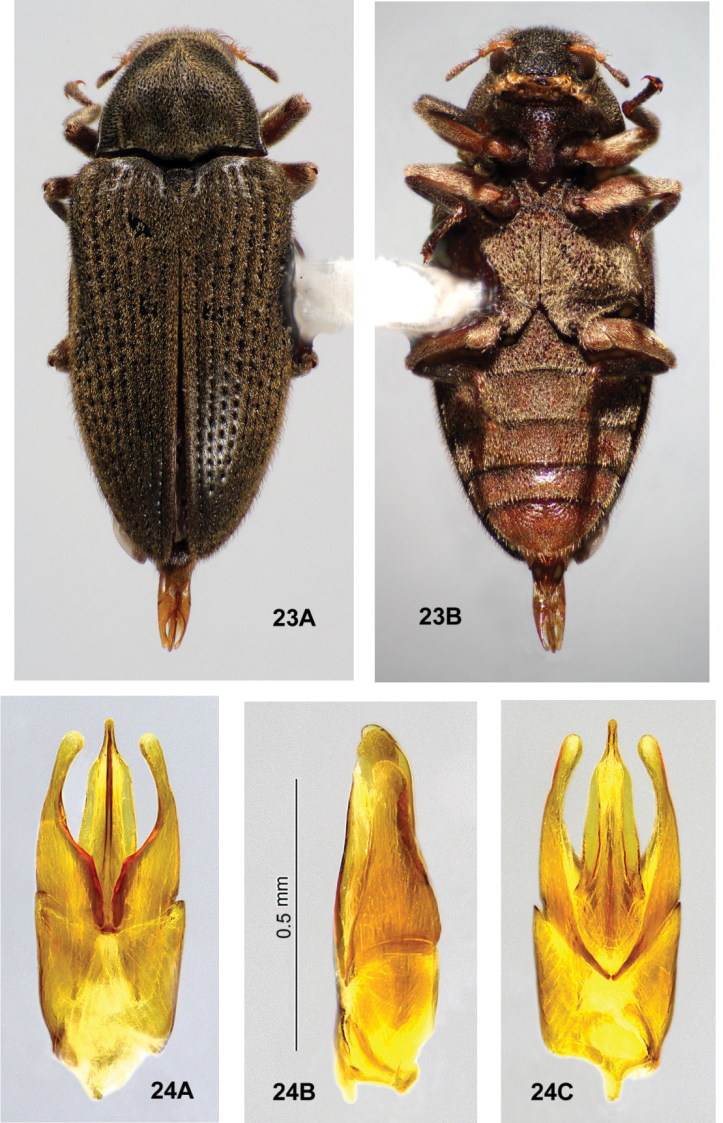
*Ovolaraaustralis*, male **23** habitus, 4.1 mm long **A** dorsal **B** ventral **24** male genitalia **A** dorsal view **B** lateral view **C** ventral view.

**Figures 25, 26.**
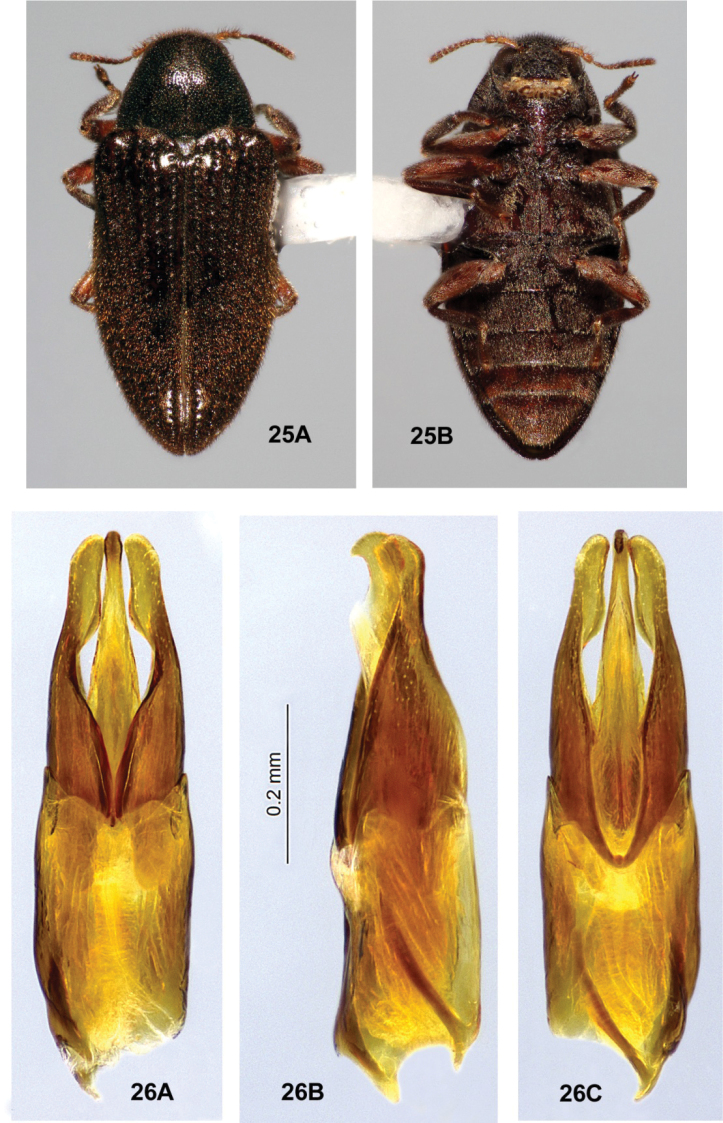
*Ovolaralawrencei* sp. nov., male **25** habitus, 3.0 mm long **A** dorsal **B** ventral **26** male genitalia **A** dorsal view **B** lateral view **C** ventral view.

Body oval or elliptical; antennae clavate, either compact or elongate; pronotum with two short, basal, sublateral carinae; pronotal disc without a transverse impression; elytra striate-punctate, each elytron with or without an accessory basal stria between striae 1 and 2, apices rounded; prosternum with a chin piece, a shelf-like, anterior extension beneath the head; prosternal process broad, with or without a distinct median longitudinal carina; mesotibiae glabrous and shiny on the posterior surfaces; apices of hind tibiae not exceeding apices of elytra; tarsi each with tarsomere 5 as long as tarsomeres 1–4 combined; abdominal ventrites 1 and 2 combined shorter than 3–5 combined (Figs 23–26, 28–31).

##### Distribution.

*Ovolara* is endemic to Australia, with four species occurring in New South Wales and Queensland (Figs 3–6)

##### Habitat and behavior.

*Ovolara* adults are most often associated with marginal or emergent stream vegetation and debris packs. Depending on the species, they may occur in areas of slow current (*O.australis*) or in fast water and rapids (*O.leai*). When captured or disturbed, *Ovolara* does not take flight as quickly as many other laraines. Specimens of all species have been collected at lights.

##### Comment.

King (1865) described the type species of the genus in *Lutochrus*, a misspelling of *Lutrochus* Erichson, 1847. Brown (1981) subsequently erected the genus *Ovolara* to include the type species, *Lutrochus* [sic] *australis* as well as *Hydrethusleai* Carter, 1926 (Brown 1981). He believed the genus to be most closely related to *Hydora*. The larva was keyed and illustrated in Glaister (1999) at the generic level.

The external morphology of the species is very similar except for that of *O.australis*. Comparison of the male genitalia is the best way to distinguish the species.

#### Ovolara australis

Taxon classificationAnimaliaColeopteraElmidae

(King, 1865)

09F7BB68-40E2-5478-99E4-CADEB38EAA93

Figs 3 , 23 , 24

##### Type locality.

Parramatta River; 33.7644°S, 151.0076°E; New South Wales, Australia (lectotype deposited in the Australian Museum, Sydney).

##### Material examined

**(114). New South Wales.** AUSTRALIA: NSW / Jerrabattgulla Creek at / Ballalaba, E Capt. Flat / 35°38'36"S,149°36'19"E / 4-I-2001, coll. C. B. Barr (9 EMEC); AUSTRALIA: NSW / 6.4 km ENE of Guthega / 7 I 2001 438 ft / Piper’s Creek (WDS-A-1357 on reverse) // William D. / Shepard, leg. (2 EMEC); AUSTRALIA: NSW / 13 km E Braidwood / 3 I 2001 / Mongarlowe River (WDS-A-1345 on reverse) // William D. / Shepard, leg. (2 EMEC); AUSTRALIA: NSW / Deua Nat Park / 4 I 2001 453' / Black lights / S35°45'00" E149°54'53" (WDS-A-1346 on reverse) // William D. / Shepard, leg. (2 EMEC); same locality; Deua River (WDS-A-1348 on reverse) (1 EMEC); Australia: N.S.W. / Paterson River Nr. / Lostock Nov. 9 /2001 G. Challet (14 EMEC); AUSTRALIA: NSW / Allyn River / 9 Nov 2001 / G. Challet, leg. (8 EMEC); AUSTRALIA: N.S.W. / Tuross River nr. / Bodalla; Nov 3 / 2001; G. Challet (6 EMEC); Mebbin St. For. / NSW 18km W of / Uki 23-24 Nov. / 1982 J.Doyen (47 ANIC, 2 EMEC); AUSTRALIA: NSW, 2km N Nana / Glen (30°6'3"S, 153°'23.6"E), 11 / November 2006, coll. D. Britton // Weedy river bank / MV lamp, Britton 2006/052 // Australian Museum / K 579954 (1 >AM); same data as for preceding; Australian Museum / K 579955 (1 >AM); 15 km NE Kyogle / At black light / 20.xi.1984 / D.J.Scambler / Australian Museum / K 579974 (1 >AM); same data as for preceding; Australian Museum / K 579975 (1 >AM); Bruxner Park, Via / Coff’s Harbour, / 25.ii.1967. N.S.W. / G. Monteith (1 QM); NSW, Eccleston 4 km / N 27/11/95 C.Watts // SAMA / 25-47747 (2 SAMA); NSW, Williams R. / nr Dungog 27/11/95 / C.Watts //SAMA / 25-47748 (1 SAMA). **Queensland.** Canungra Creek, / 4 ml. S. of Canungra, Qld / 25.XII.1974 / G. B. Monteith (9 QM); Upper Canungra Creek, / via Canungra, S.E. Qld. / 2.i.1973 / I.Naumann (1 QM); NSW [QLD], Cedar Creek / Dayboro 10 km S / 23/11/95 C.Watts // SAMA / 25-47737 (1 SAMA); Condamine R. / Killarney / 6-11-32 / H Hacker (1 QM); N. Pine R. / 23-[illegible]-32 H Hacker (1 QM); Young’s X-ing / Petrie, Q. / 2.X.59 / I.C.Yeo / (1 QM).

##### Differential diagnosis

**(n = 114).***Ovolaraaustralis* (Figs 23, 24) can be distinguished from other species of *Ovolara* by the following characters: Antennae ending in stout, moderately tight, ovoid clubs; pronotum distinctly sculptured, with a shallow, median, longitudinal sulcus at the anterior 2/3 and a broad, median, longitudinal costa at the posterior 1/3; each elytron with an accessory basal stria between striae 1 and 2; male genitalia unique. *Ovolaralawrencei* (Fig. 25), *O.leai* (Fig. 28) and *O.monteithi* (Fig. 30) have elongate antennal clubs and mostly unsculptured, smooth pronota without sulci or distinct costae; *O.lawrencei* lacks elytral accessory striae. The male genitalia of *O.lawrencei* (Fig. 26) are the most similar, but the penis of *O.australis* (Fig. 24) is abruptly constricted at the apex with the adjacent paramere apices rounded, while that of *O.lawrencei* is tapered and narrow near the apical 1/3 and the paramere inner margins are linear and clasping.

##### Variation.

The only difference observed among individuals is the degree of pronotal sculpturing, especially the depth of the median longitudinal sulcus. Measured specimens vary in size from 3.3–4.2 mm long and 1.4–1.7 mm wide (n = 30). There is little size difference between males, 3.3–4.1 mm long, 1.4–1.7 mm wide (n = 19), and females, 3.4–4.2 mm long, 1.5–1.7 mm wide (n = 11), with individuals of both at the small and large ends of the size range.

##### Distribution.

*Ovolaraaustralis* occurs in New South Wales and south Queensland, Australia (Fig. 3).

##### Habitat, behavior, and life history.

The authors found *O.australis* adults to be numerous in blackwater streams beneath undercut clumps of emergent vegetation in areas of sluggish flow. The species also has been taken at black light by the authors and other collectors. One female specimen, collected in January, was dissected and found to have 20+ eggs in her abdomen, indicating that January is within the reproductive period of the species.

##### Associated byrrhoid taxa.

Elmidae: Larainae: *Stetholuselongatus*; Elminae: *Austrolimniusmetasternalis* Carter & Zeck, *A.* spp., *Coxelmisnovemnotata* (King), *Kingolusaeratus* (Carter), *K.quatuormaculatus* (King), *K.metallicus* (King), *K.tinctus* Carter & Zeck, *K.* spp., *Notriolusmaculatus* (Carter), *N.minor* (Carter & Zeck), *N.quadriplagiatus* (Carter), *N.setosus* Carter & Zeck, *N.* spp., *Simsoniatasmanica* (Blackburn), *Simsonia* spp. Psephenidae: *Sclerocyphonstriatus* Lea.

##### Comments.

*Ovolaraaustralis*, the type species of the genus, was originally described by King (1865) in *Lutochrus*, a misspelling of *Lutrochus* Erichson, 1847; it was moved to *Ovolara* by Brown (1981). The larva of this species has been reared to the adult by Glaister (A.Glaister, in litt.).

#### Ovolara lawrencei sp. nov.

Taxon classificationAnimaliaColeopteraElmidae

8726F990-CBCD-5358-8718-D8AB6BDCA177

http://zoobank.org/6631CC2C-FA3D-4053-A72A-9ED64B97B80A

Figs 4 , 25–26 , 27

##### Type locality.

**Figure 27.**
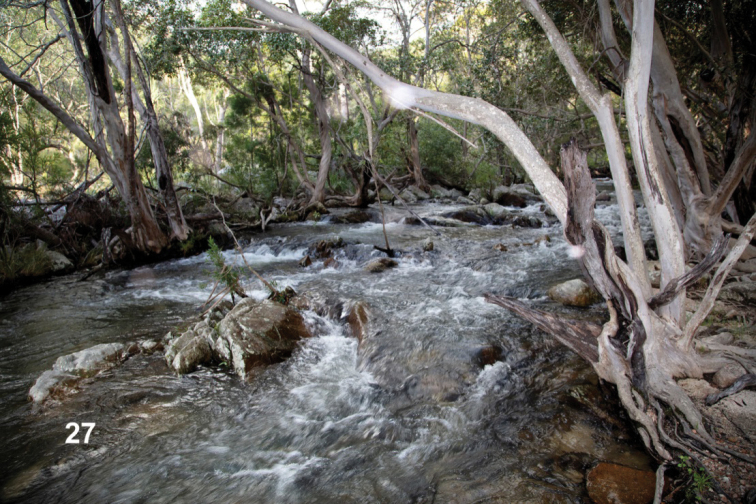
*Ovolaralawrencei* sp. nov., type locality, and *Stetholuslongipennis* sp. nov., collection locality: Emerald Creek, east of Mareeba, QLD, Australia (photograph courtesy of David Rentz, James Cook University, Smithfield, QLD).

Emerald Creek east of Mareeba; 16.9851°S, 145.4740°E; north Queensland, Australia (Fig. 27).

##### Type material.

***Holotype* male**. “AUSTRALIA: no. QLD / Emerald Creek at Hwy. 1 / E of Mareeba / 16°59’12”S, 145°28’21”E / 17-I-2001, coll. C.B. Barr // HOLOTYPE / Ovolara / lawrencei / Barr & Shepard” [red label, handwritten]. Dry pinned. Deposited in the Australian National Insect Collection, Canberra; ANIC Database Number 25-077641. ***Paratypes* (77).** Same data as for holotype (1 ANIC, 3 EMEC, 1 QM); AUSTRALIA: Queensland / Emerald Creek Store / 17 I 2001 / Emerald Creek / S16°59'12" E145°28'21" (WDS-A-1369 on reverse) // William D. / Shepard, leg. (2 EMEC); AUSTRALIA: no. QLD / Rocky Creek at Hwy. 1 / ~5 rd.km. NE of Atherton / 17°10’54”S, 145°26’59”E / 11-I-2001, coll. C.B.Barr (1 >AM, 1 ANIC, 6 EMEC); AUSTRALIA: Queensland / 5 km N Atherton / 11 I 2001 / Rocky Creek (WDS-A-1364 on reverse) // William D. / Shepard, leg. (2 EMEC); AUSTRALIA: no. QLD / Pattersons Cr. at Boar / Pocket Rd. 3 rd.km. N off / Hwy.52, SW Gordonvale / 11-I-2001, C. B. Barr // 17°12’06”S / 145°40’08”E (1 >AM, 4 EMEC, 1 QM); AUSTRALIA: Queensland / 17 km SW Gordonvale /11 I 2001 1883 ft / Patterson’s Creek (WDS-A-1365 on reverse) // William D. / Shepard, leg. (2 EMEC); AUSTRALIA: no. QLD / Bushy Creek at Hwy. 44 / just W of Julatten / 16°36’40”S, 145°20’10”E / 17-I-2001, coll. C.B. Barr (1 ANIC, 5 EMEC); AUSTRALIA: Queensland / just W of Julatten / 17 I 2001 / Bushy Creek (WDS-A-1367 on reverse) // William D. / Shepard, leg. (1 >AM, 3 EMEC, 1 QM); AUSTRALIA: no. QLD / Hunters Creek at Hwy. 44 / 5 rd. km. N Mount Molloy / 16°38’00”S, 145°19’27”E / 17-I-2001, coll. C. B. Barr (1 ANIC, 5 EMEC); AUSTRALIA: Queensland / 5 km N Mount Molloy / 17 I 2001 / Hunters Creek / S16°38’00” E145°19’27” (WDS-A-1368 on reverse) // William D. / Shepard, leg. (1 >AM, 3 EMEC, 1 QM); 17.21S 145.56E / Babinda, NQld / J.G.Brooks / without date (3 ANIC); same data as for preceding // Genitalia prep. / HO-252 ♂ / A.Calder 1997 (1 ANIC); same data as for preceding // Genitalia prep. / HO-253 ♂ / A.Calder 1997 (1 ANIC); same data as for preceding // Genitalia prep. / HO-278 ♀ / A.Calder 1997 (1 ANIC); Barron R. / Cairns, N.Q. / Apr. 1946 / J. G. Brooks // Australian Museum / K 579980 (3 >AM); Davies Creek, NQ / Oct. 1950 / J.G.Brooks // J.G. Brooks / Bequest, 1976 (2 ANIC); 15.11S 143.52E GPS / Hann River [tributary Morehead River (P. Zborowski, in litt.)] QLD / 14 Jan. 1994 at light / P.Zborowski & / E.D.Edwards (1 ANIC); 16.38S 145.19E QLD / Hunter Creek / 16 Dec. 1994 / P.Zborowski // flowing, clear stream, / sandy bottom, part / shade: rainforest (1 ANIC); Kuranda / QUEENSLAND / F. H. TAYLOR / 5-10-35 // Hydrethusleai Cart. / Genitalia prep. / HO-257 ♂ / A.Calder 1997 (1 ANIC); Kuranda, N. Qld. / 28.xii.1963 / G. Monteith // EX UQIC / DONATED / 2011 (10 QM); L’tle Mulgrave R. / N.Q. 16.xii.67 / J.G. Brooks // J.G. Brooks / Bequest, 1976 // Genitalia prep. / HO-261 ♂ / A.Calder 1997 (1 ANIC); 15.46S 144.15E GPS / Shepherd Creek QLD / 17 Jan. 1994 / water sweep / P. Zborowski, / E.D. Edwards (3 ANIC); Stewart’s Crk / Daintree, NQ / 16 Sept. 1969 / J.G. Brooks // J.G. Brooks / Bequest, 1976 (1 ANIC); same data as for preceding // Genitalia prep. / HO-258 ♂ / A.Calder 1997 (1 ANIC). Paratypes all with the following label: PARATYPE / *Ovolara* / *lawrencei* / Barr & Shepard [yellow label, printed].

##### Other material examined

**(11).** Barron Falls / QLD 12.xii.64 / J.G.Brooks (1 ANIC); locality as in preceding / 2.i.1965 / J.G.Brooks // J.G. Brooks / Bequest, 1976 // Genitalia prep. / HO-276 ♀ / A.Calder 1997 (1 ANIC); AUSTRALIA: no. QLD / Clohesy River at Hwy. 1 / 22 rd. km. NE of Mareeba / 11-I-2001, coll. C.B. Barr (1 EMEC); AUSTRALIA: Queensland / 22.2 km NE Mareeba / 11 I 2001 / Clohesy River (WDS-A-1363 on reverse) // William D. / Shepard, leg. (6 EMEC); Upper Daintree R. / Via Daintree, N.Qld. / 27.xii.1964. / G. Monteith // EX UQIC / DONATED / 2011 (1 QM). AUSTRALIA: no. QLD / Fishery Creek at / Hwy. 1, Fishery Falls / 17°11’10”S, 145°53’11”E / 18-I-2001, C. B. Barr (1 EMEC).

##### Differential diagnosis.

**Figures 28, 29.**
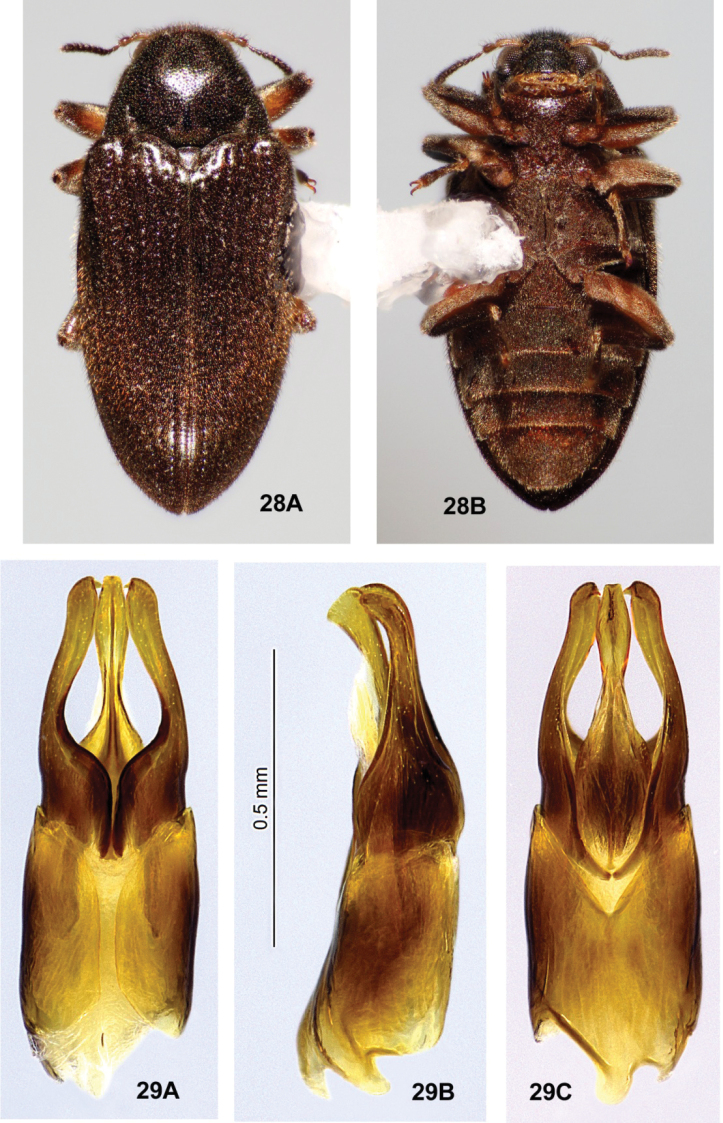
*Ovolaraleai*, male **28** habitus, 3.4 mm long **A** dorsal **B** ventral **29** male genitalia **A** dorsal view **B** lateral view **C** ventral view.

**Figures 30, 31.**
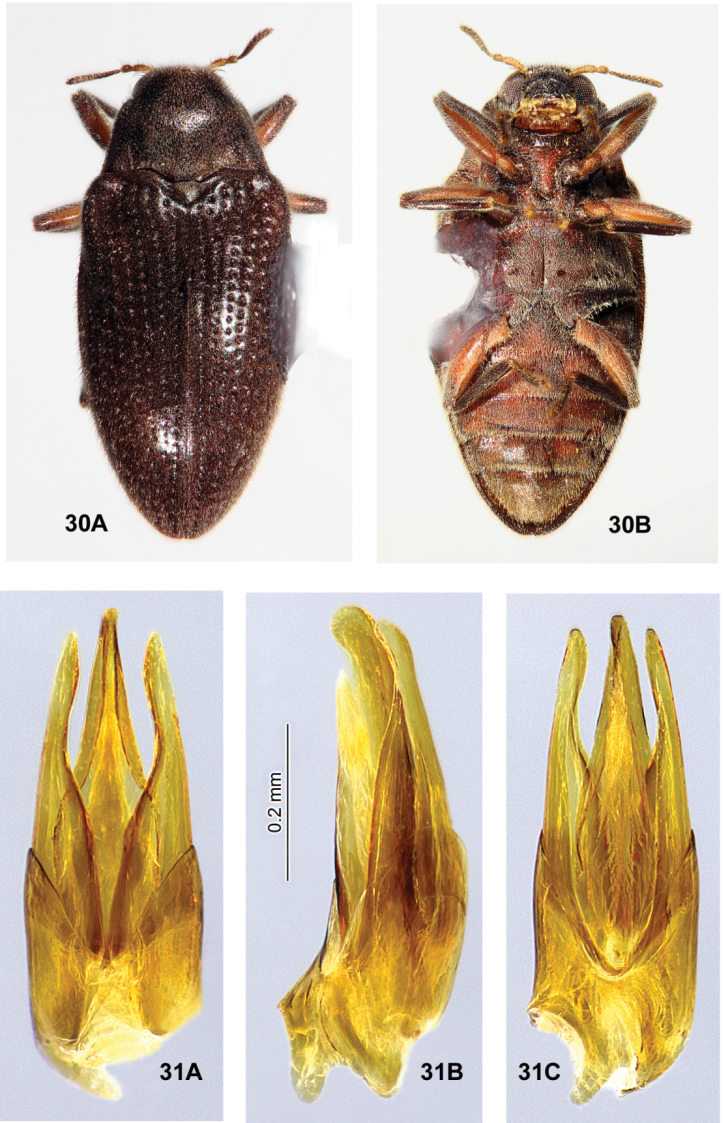
*Ovolaramonteithi* sp. nov. **30** female habitus, 3.6 mm long **A** dorsal **B** ventral **31** male genitalia **A** dorsal view **B** lateral view **C** ventral view.

*Ovolaralawrencei* (Figs 25, 26) can be distinguished from other species of *Ovolara* (Figs 23, 24, 28–31) by a combination of the following characters: Antennae clavate, elongate; pronotum mostly smooth, unsculptured, except basal margin triangularly protuberant between the prescutellar foveae; pronotal basal sublateral carinae generally shorter than the length of the scutellar shield; elytra without accessory basal striae between striae 1 and 2; and elytral punctures large and deep from base to apex. The aedeagus (Fig. 26) is unique, with the paramere inner margins linear and clasping the apical 1/3 of the tapered, narrow penis.

The other three species of *Ovolara* have elytral accessory striae of varying lengths, sometimes as short as 1–3 punctures. In addition, *Ovolaraaustralis* (Fig. 23) has an antenna with a stout, moderately tight, ovoid club; pronotum sculptured, with a distinct longitudinal sulcus and costa; and an aedeagus (Fig. 24) with the penis abruptly constricted at the apex and the adjacent paramere apices rounded. In *O.leai* (Fig. 28), the pronotal mediobasal margin is less-prominently raised; the pronotal basal sublateral carinae are as long as or longer than the scutellar shield; the apical elytral punctures are smaller and shallower than those more basal; and the aedeagus (Fig. 29) has a penis that is abruptly constricted at the middle and paramere apices that are rounded, each bearing an inner tooth. *Ovolaramonteithi* (Fig. 30) has the pronotal base flat; the pronotal basal sublateral carinae as long or longer than the scutellar shield; and the aedeagus (Fig. 31) with the lateral margins of the penis evenly convergent to an acute apex. All species, except for *O.australis*, are quite similar, and most of the above characters are somewhat variable and overlapping. Fortunately the male genitalia (Fig. 26) are distinctive and diagnostic, and are therefore the best, most reliable, identification tool.

##### Description

**(n = 89). *Body***: Size 2.9–3.3 mm long, 1.3–1.4 mm wide (n = 19). Dorsal color dark brown; head black; first two antennomeres and mouthparts yellow or yellow-brown; trochanters, basal 2/3 of femora, most of abdomen yellow-brown or red-brown; apical antennomeres, coxae, tibiae, tarsi brown. Dorsum covered with short, dense, erect and semi-erect yellow setae, cuticle shiny beneath setae; venter covered with longer, dense, semi-erect and recumbent setae. ***Head***: Densely punctate, punctures nearly contiguous. Eye weakly protruding, finely faceted, with a dorsal fringe of very long, dark setae curved over eye. Antenna with eleven antennomeres; antennomere 1 longest, arcuate, with long setae near apex; antennomere 2 subspherical, with long setae; antennomere 3 small, narrow, much longer than wide; antennomere 4 smallest; antennomeres 3–11 forming a tight, elongate club; antennomere 11 largest, apex round. Frons smooth, without impressions or carinae; frontoclypeal suture distinct, straight. Clypeus broadly rectangular, 3 × wider than long; anterior margin nearly straight; disc coarsely and densely punctate. Labrum 2 × as wide as long; disc densely punctate; anterior margin with short, dense yellow setae, anterolateral angles with dense brushes of long, yellow, curved setae. Mandible with three apical teeth; prostheca with apical setae; mola with four ridges. Maxillary palpus with four palpomeres, 3 + 4 capitate, all very setose; palpomere 1 short, annular; palpomere 2 subcylindrical, 2 × as long as wide, with tuft of long setae on medial surface; palpomere 3 conical, as long as 2 but wider; palpomere 4 conical, longer and much wider than 2, apex obliquely truncate with an oval, concave, pale yellow sensory area. Labial palpus with three palpomeres; palpomere 1 short, annular; palpomere 2 half as long as 3; palpomere 3 glabrous, rectangular, slightly longer than wide, weakly flattened, apex with oval sensory area. ***Pronotum***: Shape trapezoidal, slightly wider than long, widest at base; 0.7–0.8 mm long, 0.9–1.1 mm wide; densely punctate, punctures ~ 1 diameter apart. Anterior margin arcuate; lateral margins weakly arcuate to nearly straight, narrowly marginate; posterior margin strongly arcuate laterally, nearly straight anterior to scutellar shield; anterior angles obsolete, posterior angles almost 90°. Disc mostly smooth, slightly flattened anteromedially; two short, basal, sublateral carinae ~ 1/6 pronotal length; two small prescutellar foveae; disc anterolateral to each fovea broadly, shallowly depressed; pronotal base between prescutellar foveae protuberant. ***Scutellar shield***: Subpentagonal; anterior margin straight, apex rounded; disc flat, finely setose. ***Elytron***: 2.2–2.5 mm long, 0.7 mm wide. Elytra conjointly almost 2 × as long as wide; nearly parallel-sided from base to middle; lateral margins narrowly marginate. Elytral base usually deeply depressed between humerus and scutellar shield; disc flattened medially at 1/4 length from base; disc with ten striae, without an accessory basal stria between striae 1 and 2; striae 2, 3, 9, and 10 ending before reaching posterior margin; punctures large and deep from base to apex, spaced < 1 diameter apart; diameters smaller in rows closer to suture, becoming larger laterally; intervals slightly raised. ***Metathoracic wings***: Macropterous. ***Prosternum***: Extending anteriorly beneath head, as long anterior to procoxae as length of prosternal process; anterior margin narrowly marginate; prosternal process broad, margined, with low median longitudinal carina; process arcuate between procoxae, expanded laterally posterior to coxae, apex broadly triangular. ***Mesoventrite***: Short, wide; with a deep, broad, V-shaped mesoventral cavity to receive prosternal process; anteromedial margin raised; posterior margin nearly straight. ***Metaventrite***: Broadly rectangular, anterior margin straight; disc posteromedially depressed, laterally convex; discrimen deeply incised; short, shallow metakatepisternal suture present; disc laterally with numerous, scattered, large punctures, posteromedial depressed area devoid of punctures. ***Legs***: Setose; relatively short, similar in length, each leg with femur slightly shorter than tibia; tarsus with tarsomere 5 as long as 1–4 combined, protarsomere 5 with a single long, curved seta at dorsal apex; claws simple, slender, acute. Coxae brown, metacoxae deeply sulcate; femora with basal 3/4 yellow-brown or red-brown, apical 1/4 brown; tibiae brown, straight, mesotibiae with posterior surfaces glabrous, shiny; tarsi brown. ***Abdomen***: Five ventrites; all punctate, punctures spaced one diameter apart; ventrite 1 with equilaterally triangular intercoxal projection; ventrites 2–4 broadly rectangular; ventrites 3 and 4 each with a pair of small lobed processes on posterolateral margins and with posterior margin thickened and slightly raised; ventrite 5 densely setose; broadly triangular, lateral margins weakly arcuate to widely rounded apex. ***Aedeagus***: Phallobase, parameres and penis equally long (Fig. 26). Phallobase open dorsally, long, tubular, with parameres deeply inserted. Parameres in dorsal view (Fig. 26A) with lateral margins weakly sinuate, straight and parallel-sided in basal 1/2 then slightly converging, parallel-sided in apical 1/4; with inner margins abruptly and widely divergent, forming an enclosed, central opening; apices at apical 1/3 broadly clasping tip of penis, broadly rounded. Penis in dorsal view (Fig. 26A) with lateral margins evenly tapered to near apex, apex narrowly rounded to acute; penis laterally flattened near apex, dorsal surface with two thin, dark carinae; no visible corona; basal apophyses 1/3–1/2 as long as phallobase, straight, very broad, blunt at tips. In lateral view (Fig. 26B), penis and paramere apices broad, curved ventrally, hooked; penis apex slightly wider than paramere apex. Fibula absent. ***Ovipositor***: Well-sclerotized; elongate; baculum slightly longer than gonocoxites; proximal gonocoxite short, narrowly rectangular, curved; distal gonocoxite long and slender, medial margins nearly straight, lateral margins weakly arcuate; gonocoxites separate at bases and medially, contiguous at apices; stylus short, slender, 3 × longer than wide.

##### Variation.

Very little morphological variation was noted, except for small differences in the length of the pronotal sublateral carinae. Sizes range from 2.9–3.3 mm long and 1.3–1.4 mm wide (n = 19). The females measured are slightly larger than the males, but the female sample size is considerably smaller: females 3.0–3.3 long, 1.3–1.4 mm wide (n = 5); males 2.9–3.1 mm long, 1.3 mm wide (n = 14).

##### Etymology.

The specific epithet *lawrencei*, a noun in the genitive case, is given in honor of John F. Lawrence, arguably the most influential and prolific coleopterist of our time. An excellent review of his life and career was published by Newton et al. (2000), although somewhat prematurely because Lawrence has by no means retired.

##### Distribution.

*Ovolaralawrencei* occurs in north Queensland, Australia (Fig. 4).

##### Habitat and behavior.

*Ovolaralawrencei* was collected by the authors in small to large streams at elevations ranging from 18–654 m. All but one of these were sand-bottomed with logs and debris, some with boulders, and one had a bedrock substrate. Their waters were warm to cool, clear and colorless to brown-stained, with currents varying from sluggish to fast. At Emerald Creek (Fig. 27), the type locality at an elevation of ~ 415 m, the stream was large with a substrate of sand, gravel and boulders. *Ovolaralawrencei* specimens were found in areas of slow current among streamside vegetation, grassy margins, and debris packs, and also in faster current on logs and rocks. The beetles “played dead” in the net, remaining immobile for a period of time, and were difficult to see amongst the netted debris. The easiest method to locate them was to hold the net and debris in the water and wait for them to pop up to the surface. They did not fly readily. Specimens have been taken at light, including those collected by Monteith (G. Monteith, in litt.).

##### Associated byrrhoid taxa.

Elmidae: Larainae: *Ovolaraleai* (Carter) , *O.monteithi* sp. nov., *Potamophilinuspapuanus* Satô, *Stetholuslongipennis* sp. nov.; Elminae: *Austrolimnius* spp., *Graphelmispallidipes* (Carter), *Kingolus* spp., *Notriolustaylori* Carter & Zeck, *Notriolus* spp., *Simsonia* spp. Psephenidae: *Sclerocyphonbasicollis* Lea.

#### Ovolara leai

Taxon classificationAnimaliaColeopteraElmidae

(Carter, 1926)

B84ABC60-4565-51D5-B4E5-353AA81ED1D0

Figs 5 , 28 , 29

##### Type locality.

Cairns District; 16.9167°S, 145.7500°E; north Queensland, Australia (holotype deposited in the South Australia Museum, Adelaide). Note: The geographic coordinates given in the SAMA database place the type locality, “Cairns District,” in the middle of Cairns.

##### Material examined

**(78).** AUSTRALIA: no. QLD / Freshwater, Freshwater / Cr. at Ryan Weare Park / 16°53’13”S, 145°42’05”E / 18-I-2001, coll. C.B. Barr (3 >AM, 21 EMEC); AUSTRALIA: Queensland / Freshwater / 18 I 2001 / Freshwater Creek / S16°53'13" E145°42'05" (WDS-A-1370 on reverse) // William D. / Shepard, leg. (3 EMEC); AUSTRALIA: no. QLD / Mulgrave River at Hwy. 1 / 1 rd. km. S of Gordonvale / 17°06’10”S, 145°47’15”E / 18-I-2001, coll. C. B. Barr (3 >AM, 21 EMEC); AUSTRALIA: Queensland / 1 km S Gordonvale, 18 I 2001 94 ft / Mulgrave River / (WDS-A-1371 on reverse) // William D. / Shepard, leg. (4 ANIC, 8 EMEC); QLD. Gordonvale / Apr. 1946 / J.G.Brooks // J. G. Brooks / Bequest, 1976 (1 ANIC); same data as for preceding // Genitalia prep. / HO-277 ♀ / A.Calder 1997 (1 ANIC); Mulgrave River, QLD / at Goldsborough / 2 Jan. 1965 / J.G.Brooks (Q 148) (1 ANIC); Crystal Cascades / Cairns, N.Qld. / 30.xii.1963. / G. Monteith (6 QM); Stewarts Ck. / Daintree N.Q. / 24.9.67. J.G.B. // J. G. Brooks / Bequest, 1976 (1 ANIC); same data as for preceding / 24.ix.67 Q356 / J.G.Brooks. // J. G. Brooks / Bequest, 1976 // Ovolara sp / (needle) / det. A.Calder 1997 (1 ANIC); same data as for preceding // Genitalia prep. / HO-311 ♀ / A.Calder 1999 // *Hydrethus* / *australis* / E.B. Britton det. 1972 (1 ANIC; gold coated for SEM); Upper Daintree R. / Via Daintree, N.Qld. / 27.xii.1964. / G. Monteith (5 QM).

##### Differential diagnosis

**(n = 78).***Ovolaraleai* (Figs 28, 29) can be distinguished from other species of *Ovolara* (Figs 23–26, 30, 31) by a combination of the following characters: Antennae clavate, elongate; pronotum mostly smooth, unsculptured, with base only weakly protuberant between prescutellar foveae, if at all; pronotal basal sublateral carinae as long as or longer than the scutellar shield; elytron each with a very short, accessory basal stria of 1–3 punctures between striae 1 and 2, rarely obscure; apical elytral punctures smaller and shallower than those more basal; and the aedeagus (Fig. 29) with a penis that is abruptly constricted at the middle, and paramere apices that are rounded, each bearing an inner tooth.

*Ovolaraaustralis* (Fig. 23) has an antenna with a stout, moderately tight, ovoid club; a sculptured pronotum with a distinct longitudinal sulcus and costa; and an aedeagus (Fig. 24) with the penis abruptly constricted at the apex and the adjacent paramere apices rounded. *Ovolaralawrencei* (Fig. 25) has a pronotum with the basal margin triangularly protuberant between the prescutellar foveae; pronotal basal sublateral carinae generally shorter than the length of the scutellar shield; no elytral accessory basal striae; elytral punctures large and deep from base to apex; and unique aedeagus (Fig. 26) with the paramere inner margins linear and clasping the apical 1/3 of the tapered, narrow penis. *Ovolaramonteithi* (Fig. 30) has the pronotal base flat; apical elytral punctures large and deep; and the aedeagus (Fig. 31) with the penis lateral margins evenly convergent to an acute apex. All species, except for *O.australis*, are fairly similar externally, and the above characters are somewhat variable and overlapping. Fortunately the male genitalia (Fig. 29) are distinctive and diagnostic.

##### Variation.

Very little morphological variation was noted except for differences in the number punctures in the elytral accessory stria (1–3, rarely obscure), which sometimes varies between elytra on the same individual. Small differences in the length of the pronotal sublateral carinae were also observed. Measured specimens vary in size from 3.1–3.5 mm long and 1.4–1.5 mm wide (n = 18). The females are slightly larger than the males: females 3.3–3.5 mm long, 1.4–1.5 mm wide (n = 7); males 3.1–3.4 mm long, 1.4–1.5 mm wide (n = 11).

##### Distribution.

*Ovolaraleai* occurs in north Queensland, Australia (Fig. 5).

##### Habitat.

The authors collected this species from only two localities: Freshwater Creek at Freshwater, a large, sand-bottomed stream at an elevation of 5 m; and the Mulgrave River just south of Gordonvale, a wide, sand-bottomed river at 9 m. In both, the water was warm and clear, and the current swift. In the Mulgrave River, *O.leai* was collected from wood in rapids formed by log jams. Specimens from the QM collected by Monteith were most likely from lights (G. Monteith, in litt.).

##### Associated byrrhoid taxa.

Elmidae: Larainae: *Australaraglaisteri* sp. nov., *Ovolaralawrencei* sp. nov., *O.monteithi* sp. nov., *Potamophilinuspapuanus*, *Stetholuslongipennis* sp. nov.; Elminae: *Austrolimnius* spp., *Graphelmispallidipes*, *Kingolus* spp., *Notriolus* spp., *Simsonia* spp. Psephenidae: *Sclerocyphonbasicollis*, *S.minimus* Davis.

##### Comments.

Carter (1926) described *O.leai* in *Hydrethus* Fairmaire, 1889; it was moved to *Ovolara* by Brown (1981). The geographic coordinates for the type locality, “Cairns District,” listed in the SAMA database, place it in the middle of Cairns. The authors collected *O.leai* from Freshwater Creek only ~ 6 km northwest of Cairns.

#### Ovolara monteithi sp. nov.

Taxon classificationAnimaliaColeopteraElmidae

612898CD-3104-5862-8BDF-29086DB20D3C

http://zoobank.org/27D3BBAB-05F6-4E22-8480-C7E0F25A99E7

Figs 6 , 30 , 31

##### Type locality.

Millaa Millaa Falls Park; 17.495°S, 145.611°E; Millaa Millaa, north Queensland, Australia.

##### Type material.

***Holotype* male**. “Millaa Millaa, / 9.i.1964, N.Qld. / G. Monteith // EX UQIC / DONATED / 2011 // HOLOTYPE / Ovolara / monteithi / Barr & Shepard” [red label, handwritten]. Dry pinned. Deposited in the Queensland Museum, South Brisbane; Registration Number QM T250614. ***Paratypes* (33).** Same data as for holotype (2 EMEC, 4 QM); QLD. Gordonvale / Apr. 1946 / J.G.Brooks // J. G. Brooks / Bequest, 1976 // Genitalia prep. / HO-259 ♂ / A.Calder 1997 (1 ANIC); Henrietta Ck., / Palmerston Nat. / Pk., N.Qld. / 29.xii.1964. / G. Monteith // EX UQIC / DONATED / 2011 (2 EMEC, 6 QM); same data as for preceding; 5.xii.1965 (2 QM); same locality; Henrietta Ck., / Palmerston Nat. Pk. / 29.xii.1964. N.Qld. / H.A.Rose. / UQIC / SPECIMEN (2 QM); Stewarts Ck. / Daintree N.Q. / 24.ix.67 Q356 / J.G.Brooks. // J. G. Brooks / Bequest, 1976 // Genitalia prep. / HO-262 ♂ / A.Calder 1997 (1 ANIC); “The Boulders” Via / Babinda, N.Qld. / 15.xii.1966. / B. Cantrell // EX UQIC / DONATED / 2011 (4 QM); Upper Mulgrave / River, N.Qld. / 1-3.xii.1965. / G. Monteith // EX UQIC / DONATED / 2011 (1 QM); Upper Mulgrave River, / 30.iv.1970, N.Qld. / G. B. Monteith // EX UQIC / DONATED / 2011 (2 EMEC, 6 QM). Paratypes all with the following label: PARATYPE / *Ovolara* / *monteithi* / Barr & Shepard [yellow label, printed].

##### Differential diagnosis.

*Ovolaramonteithi* (Figs 30, 31) can be distinguished from other species of *Ovolara* (Figs 23–26, 28, 29) by a combination of the following characters: Antennae clavate, elongate; pronotum smooth, unsculptured, pronotal base flat; pronotal basal sublateral carinae as long or longer than the scutellar shield; each elytron with a short accessory basal stria of 1–3 punctures between striae 1 and 2; apical elytral punctures large and deep; and aedeagus (Fig. 31) with the penis lateral margins evenly convergent to an acute apex.

*Ovolaraaustralis* (Fig. 23) has an antenna with a stout, moderately tight, ovoid club and a sculptured pronotum with a distinct longitudinal sulcus and costa; and an aedeagus (Fig. 24) with the penis abruptly constricted at the apex and the adjacent paramere apices rounded. *Ovolaralawrencei* (Fig. 25) has the pronotal basal margin protuberant between the prescutellar fovea; the pronotal basal sublateral carinae generally shorter than the length of the scutellar shield; no elytral accessory striae; and a unique aedeagus (Fig. 26) with the paramere inner margins linear and clasping the apical 1/3 of the tapered, narrow penis. In *O.leai* (Fig. 28), the apical elytral punctures are smaller and shallower than those more basal; and the aedeagus (Fig. 29) has a penis that is abruptly constricted at the middle, and paramere apices that are rounded, each bearing an inner tooth. All species, except for *O.australis*, are fairly similar externally, and the above characters are somewhat variable and overlapping. Fortunately the male genitalia (Fig. 31) are distinctive and diagnostic.

##### Description

**(n = 34). *Body***: Size 2.9–3.6 mm long, 1.2–1.5 mm wide (n = 11). Dorsal color medium to dark brown; head black; first two antennomeres, trochanters, basal 3/4 of femora yellow or yellow-brown; tibiae brown or black; apical antennomeres, tarsi brown; venter including coxae yellow-brown or red-brown. Dorsum covered with short, dense, erect and semi-erect, pale yellow setae, cuticle shiny beneath setae; venter covered with longer, dense, semi-erect and recumbent setae. ***Head***: Densely punctate, punctures < 1 diameter apart, sometimes nearly contiguous. Eye weakly protruding, finely faceted, with a dorsal fringe of long setae curved over eye. Antenna with eleven antennomeres; antennomere 1 elongate, nearly cylindrical, arcuate, with long setae near apex; antennomere 2 subspherical with long, curved setae; antennomere 3 elongate, narrow; antennomere 4 smallest; antennomeres 3–11 forming a tight, elongate club; antennomere 11 largest, apex round. Frons smooth, without impressions or carinae; frontoclypeal suture distinct, weakly arcuate. Clypeus broadly rectangular, 3 × wider than long, anterior margin arcuate; disc coarsely punctate. Labrum 2 × wider than long; disc punctate; anterior margin with short, dense, pale yellow setae, anterolateral angles with dense brushes of long, yellow, curved setae. Maxillary palpus with four palpomeres, 3 + 4 capitate, all very setose; palpomere 1 annular, short; palpomere 2 fusiform, 2 × as long as wide, with tuft of long setae on medial surface; palpomere 3 asymmetrical, wider than long; palpomere 4 subovoid, longer and wider than 2, apex obliquely truncate with an oval, pale yellow sensory area. Labial palpus with three palpomeres; palpomere 1 short, annular; palpomere 2 elongate, narrow; palpomere 3 glabrous, rectangular, flattened, much wider than palpomere 2, apex truncate with oval sensory area. ***Pronotum***: Shape trapezoidal, wider than long, widest at base; 0.7–0.9 mm long, 0.9–1.2 mm wide; densely, finely punctate, punctures 1.0–1.5 diameters apart. Anterior margin arcuate; lateral margins nearly straight, narrowly marginate; posterior margin strongly arcuate laterally, straight anterior to scutellar shield; anterior angles obsolete, posterior angles almost 90°. Disc mostly smooth, slightly flattened; two basal, sublateral carinae as long as 1/4 pronotal length or shorter; disc shallowly depressed around bases of carinae; two small prescutellar foveae, anterolateral disc slightly depressed or not. ***Scutellar shield***: Subtriangular; disc weakly convex, finely setose. ***Elytron***: 2.2–2.7 mm long, 0.6–0.8 mm wide. Elytra conjointly almost 2 × as long as wide, widest at 1/2 distance from base; lateral margins narrowly marginate. Humerus inflated, moderately prominent; elytral base depressed between humerus and scutellar shield; disc evenly convex, with ten striae and a very short, accessory, basal stria of 1–3 punctures between striae 1 and 2; striae 2, 3, 9, and 10 ending before reaching posterior margin; punctures deep and moderately large from base to apex, diameters smaller in rows closer to suture, becoming larger laterally; intervals mostly flat. ***Metathoracic wings***: Macropterous. ***Prosternum***: Extending anteriorly beneath head, shorter anterior to procoxae than length of prosternal process; anterior margin narrowly marginate; prosternal process broad, widely margined, with a low, rounded, median longitudinal carina; process arrowhead-shaped, narrowed and arcuate between procoxae, expanded laterally posterior to coxae, broadly triangular at apex, tip rounded. ***Mesoventrite***: Short, wide; with a deep, broad, U-shaped mesoventral cavity to receive prosternal process; anteromedial margin raised; posterior margin nearly straight. ***Metaventrite***: Broadly rectangular, anterior margin straight; disc posteromedially depressed, laterally convex; discrimen more deeply incised posteriorly than anteriorly; metakatepisternal suture shallow; disc laterally with irregularly spaced, large punctures, medially devoid of punctures. ***Legs***: Setose; relatively short, similar in length, each leg with femur slightly shorter than tibia; tarsus with tarsomere 5 as long as 1–4 combined, protarsomere 5 with a single long, curved seta at dorsal apex; claws simple, short, slender, acute. Coxae yellow-brown or red-brown, metacoxae deeply sulcate; femora with basal 3/4 yellow or yellow-brown, apical 1/4 brown; tibiae brown or black, straight; mesotibiae with posterior surfaces flat, glabrous, shiny; tarsi brown. ***Abdomen***: Five ventrites; all punctate, punctures spaced one diameter apart; ventrite 1 with equilaterally triangular intercoxal projection; ventrites 2–4 broadly rectangular, each with a pair of small lobed processes on posterolateral margins; ventrites 3 and 4 with posterior margin thickened and slightly raised; ventrite 5 densely setose, slightly flattened, broadly triangular, lateral margins weakly curved to widely rounded apex. ***Aedeagus***: Phallobase short, shorter than parameres and penis; penis slightly longer than parameres (Fig. 31). Phallobase open dorsally with parameres deeply inserted. Parameres in dorsal view (Fig. 31A) widest basally, narrowest at apical 1/3; lateral margins gradually convergent; medial margins gradually divergent in basal 2/3, moderately arcuate in apical 1/3, apices narrowly rounded. Penis in dorsal view (Fig. 31A) with lateral margins evenly convergent to acute apex; penis laterally flattened near apex, dorsolateral margins with two thin, dark carinae; no visible corona; basal apophyses long, 2/3–3/4 as long as phallobase, straight, broad, blunt at tips. Paramere in lateral view (Fig. 31B) subtriangular at basal 2/3, dorsal margin weakly arcuate, ventral margin nearly straight; narrowed abruptly at apical 1/3, apex curved ventrally, rounded at tip. Penis in lateral view (Fig. 31B) with apex curved ventrally, tip broadly rounded, wider than paramere tip. Fibula absent.

##### Variation.

Very little morphological variation was noted except for differences in the number of punctures (1–3) in the elytral accessory striae, which is sometimes variable between elytra on the same individual. Differences were also observed in the length of the pronotal sublateral carinae which can be up to 1/4 the length of the pronotum or shorter. Measured specimens vary in size from 2.9–3.6 mm long and 1.2–1.5 mm wide (n = 11). The sizes of the males and females overlap, but the females are generally larger than the males: females 3.2–3.6 long, 1.3–1.5 mm wide (n = 6); males 2.9–3.4 mm long, 1.2–1.4 mm wide (n = 5).

##### Etymology.

The specific epithet *monteithi*, a noun in the genitive case, is given in honor of Geoffrey Monteith of the Queensland Museum who has collected > 200,000 insects, including nearly all of the specimens of Elmidae housed there.

##### Distribution.

*Ovolaramonteithi* occurs in north Queensland, Australia (Fig. 6).

##### Habitat.

The specimens collected at the type locality, Millaa Millaa Falls Park on the Atherton Tableland, were taken at mercury vapor light near a large waterfall at 780 m elevation (G. Monteith, in litt.) . The other six collection localities included streams and small rivers in rainforest, remnant rainforest, and farmland habitats at elevations from 20–850 m. Most of the QM specimens were collected at mercury vapor lights near streams and rivers (G. Monteith, in litt.).

##### Associated byrrhoid taxa.

Elmidae: Larainae: *Australaraglaisteri* sp. nov., *Ovolaralawrencei* sp. nov., *O.leai*, *Potamophilinuspapuanus*, *Stetholuslongipennis* sp. nov.

#### Genus Potamophilinus

Taxon classificationAnimaliaColeopteraElmidae

Grouvelle, 1896

DA7688BC-237D-52A6-B61E-6ECA3AE98216

##### Type species.

*Potamophiluslongipes* Grouvelle, 1892.

##### Differential diagnosis.

Pronotum with a wide, U-shaped, transverse impression at the anterior third, without basal sublateral carinae; pronotal posterior angles blunt, not distinctly bidentate; elytral apices angulate; prosternal process carinate, broad between procoxae, abruptly narrowed and spinose between mesocoxae, acuminate apically; apices of metatibiae exceeding apices of elytra; abdominal ventrites 1+2 longer than 3+4+5, ventrite 1 very long, ventrite 2 long, ventrites 3–5 each very short, loosely fitted to epipleura. *Potamophilinus* is easily differentiated from all other Australian laraine genera by the above characteristics of the pronotum, elytral apices and prosternal process. Although *Potamophilus* Germar and *Parapotamophilus* Brown have not been reported from Australia, like *Potamophilinus* they occur in Papua New Guinea and therefore are being included here in the generic diagnosis. *Potamophilus* differs by having the pronotal posterior angles acute, distinctly bidentate; elytral apices acute, divergent; apices of metatibiae just reaching apices of elytra; abdominal ventrites 1+2 shorter than ventrites 3+4+5. *Parapotamophilus* has the pronotum without a transverse impression; elytral apices rounded; prosternal process broad, not spinose; abdominal ventrites 1+2 shorter than ventrites 3+4+5.

##### Distribution.

Thirteen species of *Potamophilinus* occur from eastern Asia to Australia.

##### Comments.

Grouvelle (1896) erected *Potamophilinus* and designated *Potamophiluslongipes* Grouvelle, 1892, as the type species.

In his unpublished checklist of elmid species, Calder (1992) listed an undescribed species of *Potamophilinus* from north Queensland based on three specimens in ANIC labeled “W. Claudie River / Iron Range, NQ / 13 May 1971 / J.G.Brooks”. We examined the specimens and concluded that they are *P.papuanus* Satô, described from New Guinea, by comparison with paratypes of that species, the original description, and the male genitalia. Lawrence and Britton (1994) first reported the genus from Australia, probably from Calder’s determination. As mentioned in the differential diagnosis, two other genera of Larainae besides *Potamophilinus* occur in nearby Papua New Guinea, *Potamophilus* and *Parapotamophilus*. Possibly they too will be found in Australia in the future. Glaister (1999) keyed and illustrated larvae from the Northern Territory which she assumed to be *Potamophilinus*, but this was not verified by rearing to adult.

#### Potamophilinus papuanus

Taxon classificationAnimaliaColeopteraElmidae

Satô, 1973

4EC603F0-D0AF-58B7-A2B8-174B73146A08

Figs 7 , 32 , 33

##### Type locality.

Wum, Upper Jimi Valley, NE New Guinea (Papua New Guinea) (holotype deposited in the Bishop Museum, Honolulu, Hawaii). Geographic coordinates unavailable.

**Paratypes examined (2).** NEW GUINEA (NE) / Wum, Upper Jimmi [Jimi] V. / 840 m. VII-17-'55 // J.L. Gressitt / Collector // Paratype / Potamophilinus / papuanus M. Sato / DET. M. SATO 1972 (1 EMEC); NEW GUINEA (NE) / Wau, Morobe Distr. / 1200 m, 25-30.IV.62 // Light Trap / J. Sedlacek / BISHOP // Paratype / Potamophilinus / papuanus M. Sato / DET. M. SATO 1972 (1 EMEC).

**Other material examined (13).** AUSTRALIA: no. QLD / Freshwater, Freshwater / Cr. at Ryan Weare Park / 16°53’13”S, 145°42’05”E / 18-I-2001, coll. C.B. Barr (2 EMEC); AUSTRALIA: Queensland / Freshwater / 18 I 2001 / Freshwater Creek / S16°53'13" E145°42'05" (WDS-A-1370 on reverse) // William D. / Shepard, leg. // Potamophilinus / papuanus / W. D. Shepard (1 ANIC, 4 EMEC); AUSTRALIA: Queensland / Emerald Creek Store / 17 I 2001 / Emerald Creek / S16°59'12" E145°28'21" (WDS-A-1369 on reverse) // William D. / Shepard, leg // Potamophilinus / papuanus / W. D. Shepard (2 EMEC); Upper Daintree R. / Via Daintree, / 27.xii.1964. N.Qld. / G. Monteith // EX UQIC / DONATED / 2011 (1 QM); W. Claudie River / Iron Range, NQ / 13 May 1971 / J.G.Brooks (3 ANIC).

##### Differential diagnosis

**Figures 32, 33.**
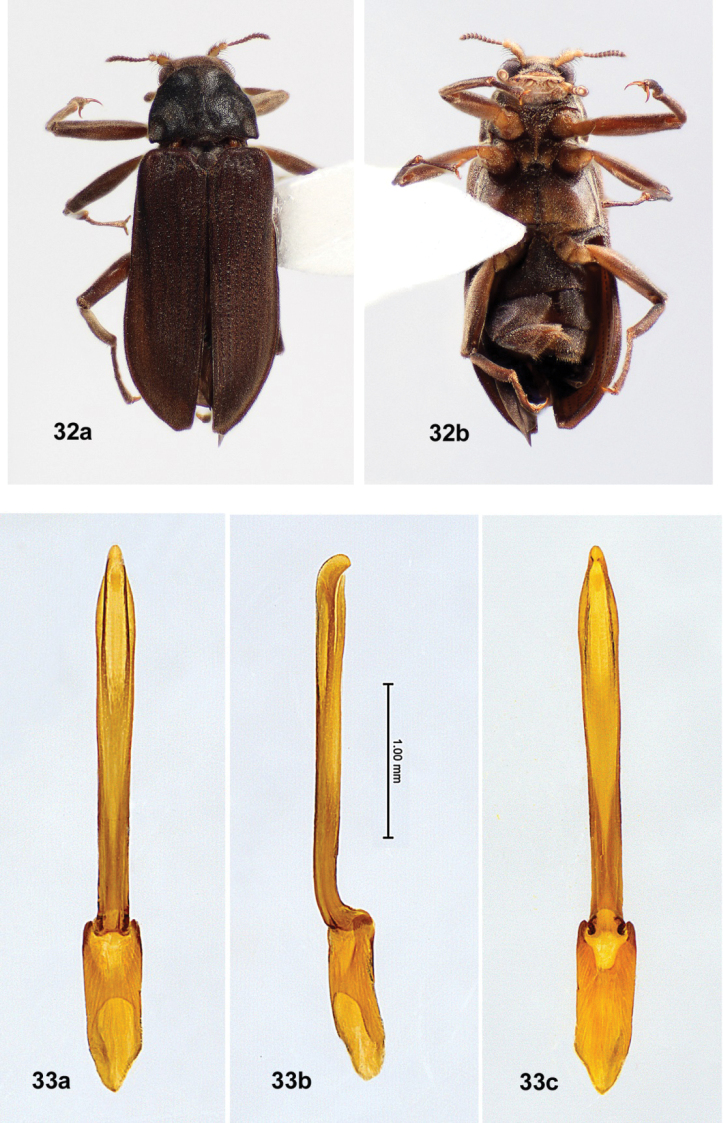
*Potamophilinuspapuanus*, male **32** habitus, 4.6 mm long **A** dorsal **B** ventral **33** male genitalia **A** dorsal view **B** lateral view **C** ventral view.

**(n = 15).** Body (Fig. 32) elongate, sides subparallel; antennae loosely clavate, not reaching to middle of pronotum; maxillary palpi each with tip of palpomere 4 obliquely truncate, elliptical; labial palpi with tip of palpomere 3 truncate, oval; pronotum flat, with a wide, U-shaped, transverse impression at anterior 1/3, without basal sublateral carinae; pronotal anterior angles depressed, posterior angles blunt, each with a large, adjacent oval depression; elytra striate-punctate, apices angulate; pro- and mesofemora broad and anteriorly flattened to slightly concave; prosternal process very long, carinate, broad between procoxae, abruptly narrowed and spinose between mesocoxae, apex acuminate; apices of metatibiae exceeding apices of elytra; abdomen with six visible ventrites, loosely fitted to epipleura; aedeagus (Fig. 33) very long and slender; penis and parameres abruptly angled at base; parameres fused with penis basally and appressed apically. *Potamophilinuspapuanus* (Fig. 32) is easily differentiated from all other Australian laraines by characteristics of the pronotum, elytral apices, prosternal process, and unusual male genitalia (Fig. 33).

##### Variation.

Measured specimens from Australia vary in size from 4.4–4.9 mm long and 1.7–2.1 mm wide (n = 11). The females are larger than the males, but the sample size is small: females 4.6–4.9 mm long, 1.8–2.1 mm wide (n = 6); males 4.4–4.7 mm long, 1.7–1.9 mm wide (n = 5). In the species description, Satô (1973) reported a much wider size range in Papua New Guinea specimens: 4.2–5.6 mm long, 1.6–2.1 mm wide. In measurements of two specimens from the type series, the male is a full millimeter shorter than the female. The species is sexually dimorphic with males having the elytral apices truncate and angulate, and females having the elytral apices broadly rounded except for each with a deflexed, triangular tooth near the inner margin (visible in posterior view).

##### Distribution.

*Potamophilinuspapuanus* occurs in Papua New Guinea, north Queensland (Fig. 7) and possibly the Northern Territory (Glaister 1992, 1999), Australia.

##### Habitat and life history.

Our few records of *P.papuanus* are from large, sand-bottomed creeks with warm, clear water where specimens were collected from logs and branches in fast current. In the NT, *Potamophilinus* larvae occur among matted roots at margins of sandy streams (A.Glaister, in litt.). Three adult females, all collected in January, were dissected in the lab and had eggs in their abdomens: two had four eggs each and one had > 20 eggs. Therefore, January is within the reproductive period of the species. One of the specimens examined from New Guinea was collected in a light trap, as was the QM specimen although not labeled as such (G. Monteith, in litt.) .

##### Associated byrrhoid taxa.

Elmidae: Larainae: *Ovolaralawrencei* sp. nov., *O.leai*, *O.monteithi* sp. nov., *Stetholuslongipennis* sp. nov.; Elminae: *Austrolimnius* spp., *Graphelmispallidipes*, *Notriolustaylori*, *Notriolus* spp., *Simsonia* spp.

##### Comments.

*Potamophilinuspapuanus* was described from Papua New Guinea, and its occurrence in Australia is not listed in the world elmid catalog by Jäch et al. (2016). In order to confirm the species identification we examined two paratypes of *P.papuanus* from New Guinea and compared Satô’s illustration of the male genitalia (Satô 1973) with the genitalia of four Australian specimens from north Queensland.

#### Genus Stetholus

Taxon classificationAnimaliaColeopteraElmidae

Carter & Zeck, 1929

3CE1AA6B-5B97-5623-ABB1-706CC1214B3E

##### Type species.

*Stetholuselongatus* Carter & Zeck, 1929.

##### Diagnosis.

**Figures 34, 35.**
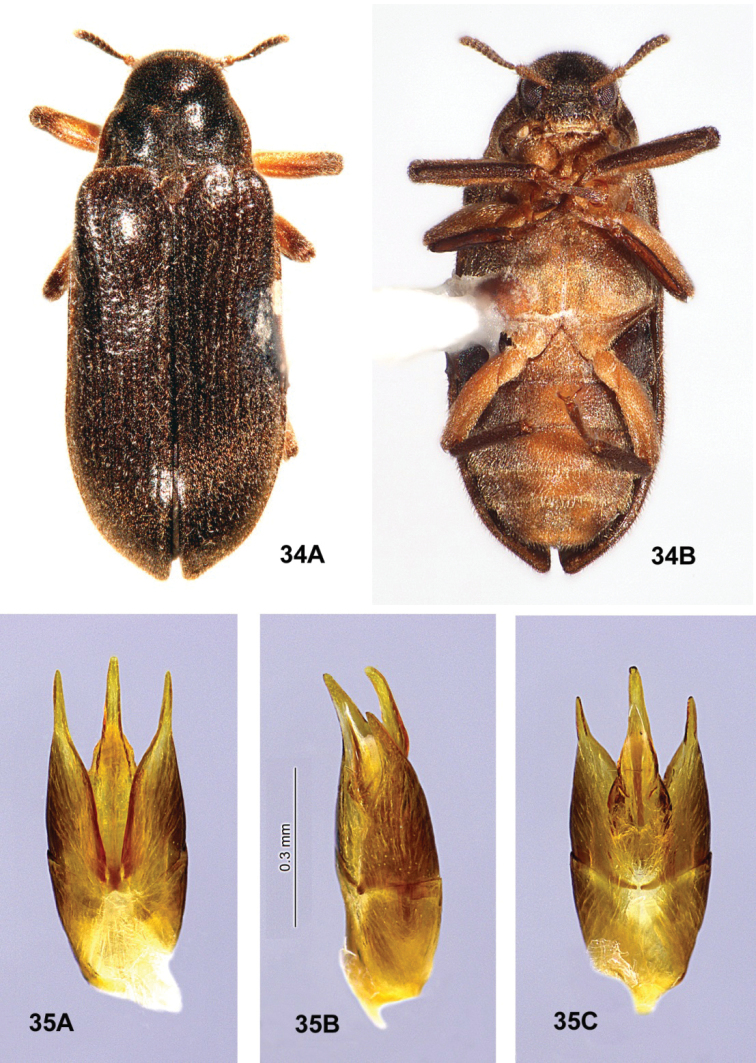
*Stetholuscarinatus* sp. nov., holotype male **34** habitus, 3.7 mm long **A** dorsal **B** ventral **35** male genitalia **A** dorsal view **B** lateral view **C** ventral view.

**Figures 36, 37.**
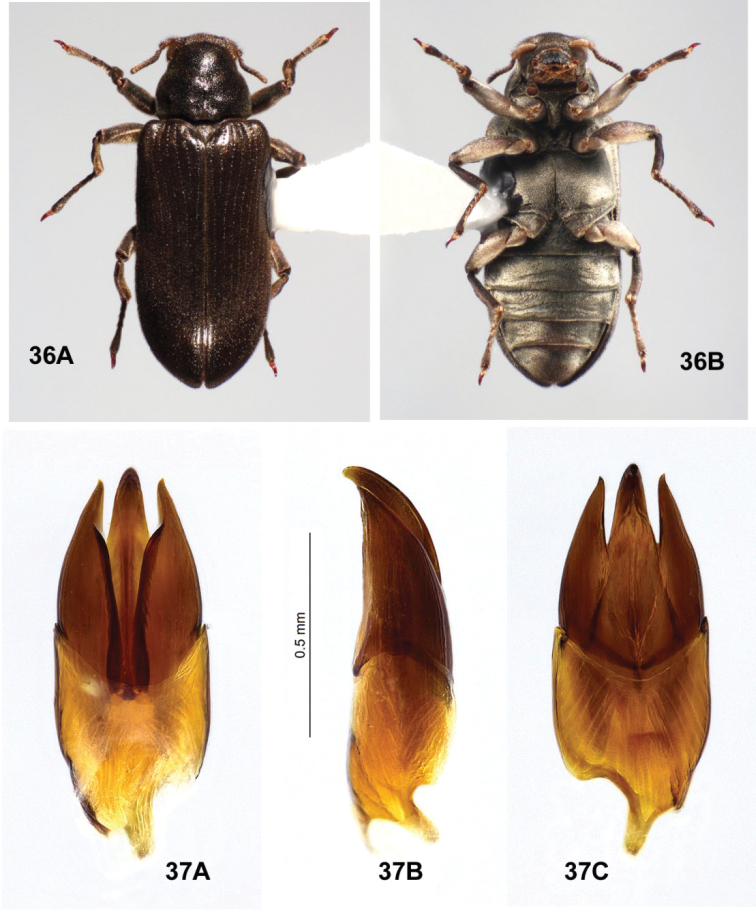
*Stetholuselongatus***36** female habitus, 5.2 mm long **A** dorsal **B** ventral **37** male genitalia **A** dorsal view **B** lateral view **C** ventral view.

**Figures 38, 39.**
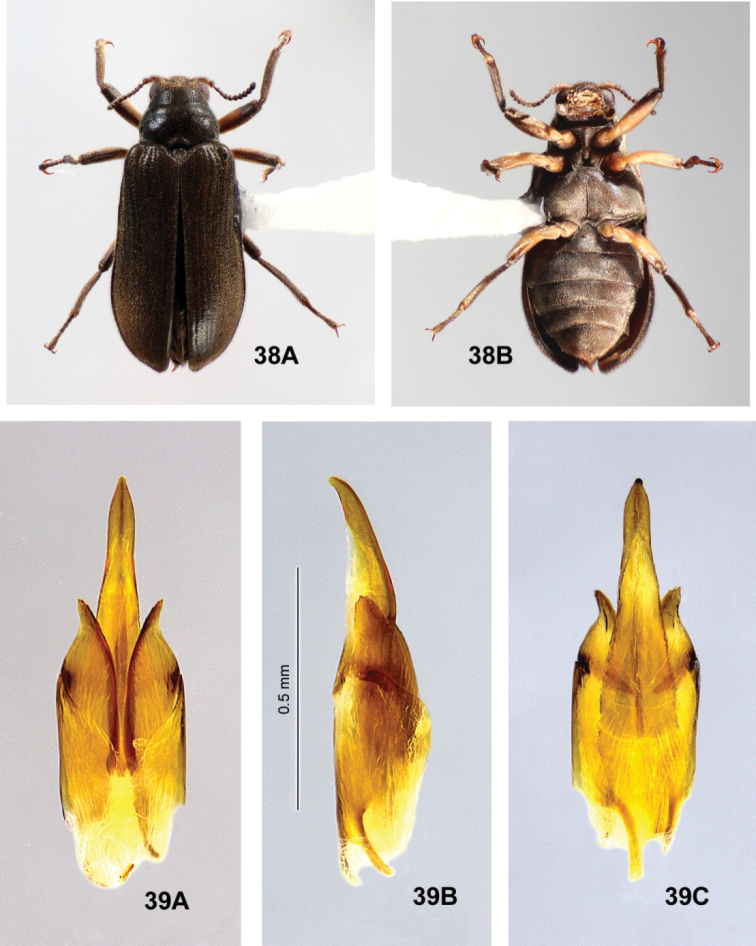
*Stetholuslongipennis* sp. nov., male **38** habitus, 4.2 mm long **A** dorsal **B** ventral **39** male genitalia **A** dorsal view **B** lateral view **C** ventral view.

**Figure 40.**
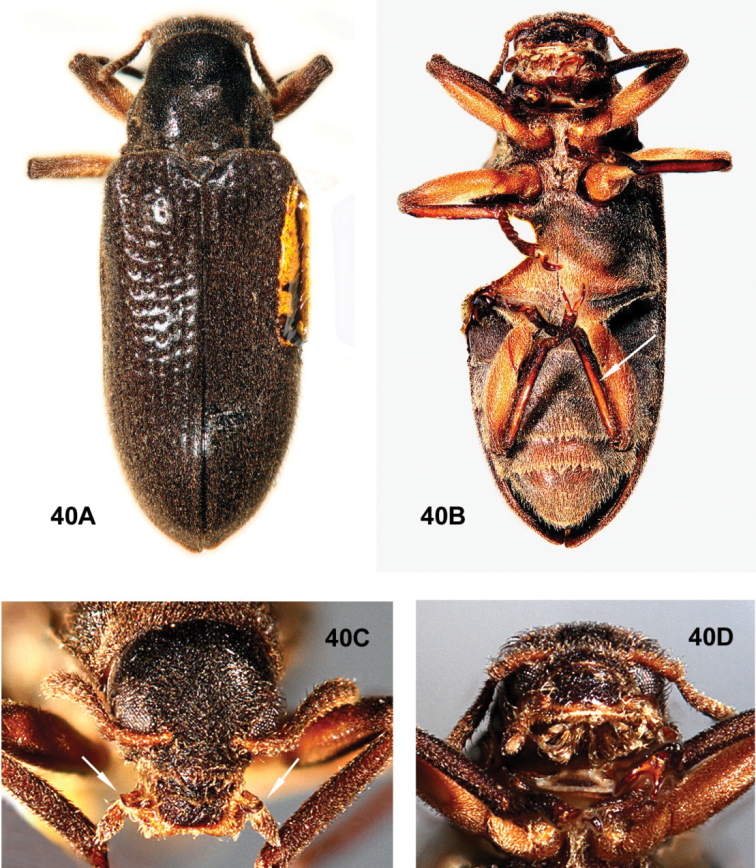
*Stetholusmetatibialis* sp. nov., holotype female, 3.9 mm long **A** dorsal habitus **B** ventral habitus **C** head, frontal view **D** head, ventral view.

**Figures 41, 42.**
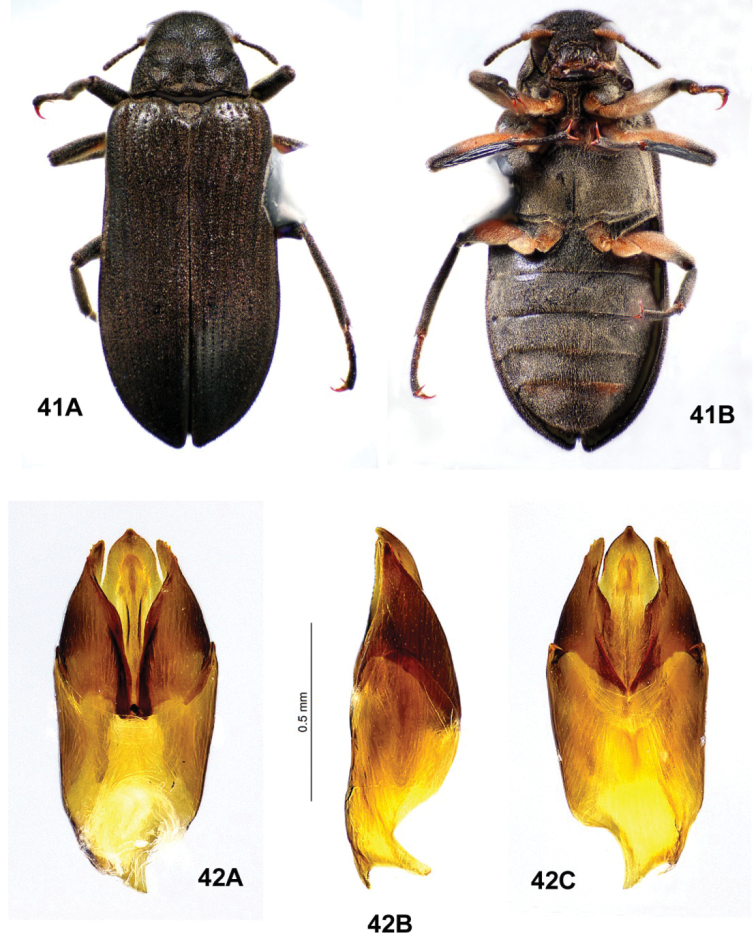
*Stetholusworonora* sp. nov., holotype male **41** habitus, 5.2 mm long **A** dorsal **B** ventral **42** male genitalia **A** dorsal view **B** lateral view **C** ventral view.

Body elongate, sides subparallel; antennae clavate, either compact or elongate, reaching at least to middle of pronotum; labrum with lateral brushes of long, curved setae; maxillary palpi long, prominent, enlarged apically, each with nearly half of palpomere 4 composed of a ventral, widely open, white sensory area obliquely angled from the apex to the base; pronotum with or without basal sublateral carinae; pronotal disc with a shallow to moderately deep, transverse, broadly V-shaped impression generally at anterior 1/3–1/2; elytra striate-punctate, laterally compressed at basal 1/2, apices rounded; prosternum very short anterior to procoxae; prosternal process moderately narrow, with a median longitudinal carina; apices of hind tibiae not exceeding apices of elytra; abdominal ventrites 1–2 combined shorter than 3–5 combined (Figs 34–42).

##### Distribution.

*Stetholus* is endemic to Australia, with species occurring in Queensland, the Australian Capital Territory, New South Wales and Victoria (Figs 8–12). There was a record in the Atlas of Living Australia (ALA) https://www.ala.org.au/ database of a specimen from Tasmania, but the specimen was misidentified therefore the record was erroneous (S. Grove, in litt.). It has since been deleted.

##### Habitat and behavior.

Adults are usually found in fast or turbulent water in rocky chutes, below waterfalls and spillways, on log jams and boulders in rapids, and among root masses in the current, often in large aggregations. They fly readily when disturbed. Specimens also have been collected with light traps and flight intercept traps (A.Glaister, in litt.; G. Monteith, in litt.).

##### Comments.

Three of the five known species exhibit secondary sexual dimorphism with the females having the posterior 1/4 of the elytron slightly explanate lateral to stria 11. This is most pronounced in *S.longipennis* sp. nov., but is less so and somewhat variable in *S.elongatus* and *S.woronora* sp. nov. The larva of *Stetholus* was keyed and illustrated by Glaister (1999).

#### Stetholus carinatus sp. nov.

Taxon classificationAnimaliaColeopteraElmidae

70A451A0-211F-543F-8A35-AF2D7D5EB895

http://zoobank.org/602C85EB-0E6E-4893-9087-F2F1F0ACB765

Figs 8 , 34 , 35

##### Type locality.

Upper North Creek, Mt. Elliot, Bowling Green Bay National Park southeast of Townsville; 19.490° S, 146.974° E; north Queensland, Australia.

##### Type material.

***Holotype* male.** “Mt Elliot NP, N.E.QLD / (Upper North Ck, 1000m) / 3-5 Dec 1986 / Monteith, Thompson&Hamlet / Flight intercept trap // HOLOTYPE / Stetholus / carinatus / Barr & Shepard” [red label, handwritten]. Dry pinned. Deposited in the Queensland Museum, South Brisbane; Registration Number QM T250616.

##### Differential diagnosis.

The single male specimen of *S.carinatus* (Figs 34, 35) is characterized by the following: shorter (3.7 mm) than other *Stetholus* species (3.9 mm or longer) (Figs 36–42); pronotum with a pair of distinct, long, basal sublateral carinae; elytron with a short, faint, accessory basal stria with a few punctures between striae 1 and 2; mesotibiae with posterior surfaces glabrous and shiny, metatibiae entirely setose; male genitalia unique (Fig. 35) (those of *S.metatibialis* are unknown). The species is separated from all other *Stetholus* except *S.metatibialis* (Fig. 40), which it most closely resembles, by the long, basal sublateral pronotal carinae. *Stetholuscarinatus* differs from *S.metatibialis* as follows: length shorter (3.7 mm vs. 3.9 mm); metatibiae entirely setose; elytron with accessory stria obscure.

##### Description

**(n = 1)**. **Holotype male. *Body***: Size 3.7 mm long, 1.4 mm wide; elongate, ~ 2 × longer than wide. Dorsal color dark brown; head black; first two antennomeres, palpi, venter, coxae, trochanters, femora yellow or yellow-brown. Short yellow setae on all surfaces. ***Head***: Densely and finely punctate, punctures < 1 diameter apart or nearly contiguous; densely setose. Vertex with a faint V-shaped impression, open anteriorly, extending from antennal bases towards occiput; frontoclypeal suture arcuate. Antenna with eleven antennomeres; antennomeres 1 and 2 yellow-brown with long, coarse, dark setae; antennomere 1 longest, ~ 3 × longer than wide, curved; antennomere 2 spherical; antennomeres 3–11 brown with dense yellow setae, subserrate, together forming an elongate club; antennomeres 7–11 of equal width, antennomere 11 short with bluntly rounded apex. Eye finely faceted, suboval at base, not protuberant; fringe of long, curved, black setae at dorsal margin. Clypeus convex, broadly rectangular, weakly emarginate; disc densely setose, anterior and lateral margins with long setal fringe. Labrum rectangular, longer and slightly narrower than clypeus; setose; anterior margin emarginate with a band of short, yellow setae; lateral margins with dense fringes of long, yellow setae, each margin with a discrete tuft of longer, darker, curved setae (setal origin unclear, possibly mandibular). Maxillary palpus yellow, with four setose, palpomeres; palpomere 1 short, annular; palpomere 2 twice as long as wide; palpomere 3 nearly as long as 2, wider apically; palpomere 4 wide, ovoid, ventral surface with a broadly oval, slightly concave, white sensory area angled obliquely from the apex to the base. Labial palpus yellow, glabrous, with three palpomeres; palpomeres 1 and 2 short, annular; palpomere 3 broadest, apex truncate with a narrowly oval, flat, white sensory area. *P****ronotum***: Shape generally trapezoidal, wider than long, widest at base; 0.9 mm long, 1.1 mm wide; disc densely punctate, punctures spaced < 1 diameter apart. Anterior margin arcuate; anterior angles obsolete; lateral margins weakly sinuate, moderately explanate at basal 2/3; posterior angles 90°, sharp, widely excavated; posterior margin weakly trisinuate. Disc weakly convex with a shallow, transverse V-shaped impression at apical 1/3; two basal, sublateral carinae 1/3–1/2 as long as pronotum, bordered by impressions, medial impressions shallow, elongate; two small, shallow prescutellar foveae. ***Scutellar shield***: Longer than wide, apex rounded; flat; densely setose. ***Elytron***: 2.8 mm long, 0.7 mm wide. Elytra conjointly 2 × as long as wide; anterior 2/3 parallel-sided; posterior 1/3 widest; lateral margins narrowly marginate. Humerus inflated, elytral base depressed medially; disc weakly convex at anterior 1/4 then flattened. Disc with ten punctate, weakly impressed striae, intervals flat; accessory basal stria between striae 1 and 2 short, faint, with few punctures; punctures of striae 2 and 3 very small and obscure near base; striae 3 and 4 join near apex; disc punctures mostly separated by one diameter, smaller apically. ***Metathoracic wings***: Macropterous. ***Prosternum***: Very short anterior to procoxae. Prosternal process very narrow, long, 4 × longer than wide; parallel-sided posterior to coxae; apex narrowly rounded; surface tomentose. ***Mesoventrite***: Short, very setose, with a deep mesoventral cavity to receive prosternal process. ***Metaventrite***: Broadly rectangular; very setose; posterior 1/2–2/3 with a moderately wide, shallow, median depression, laterally convex; discrimen extending almost from anterior to posterior margin, narrowly incised at posterior 1/2; metakatepisternal suture distinct; disc laterally with shallow, closely spaced punctures; medially punctures mostly obscured by a broad, triangular patch of long, dense, recumbent, yellow setae. ***Legs***: Of similar lengths; each leg with femur and tibia subequal in length; tarsus with tarsomere 5 longer than tarsomeres 1–4 combined; claws simple, long, sharply acute. Coxae yellow, metacoxae deeply sulcate; femora yellow, dorsal surfaces of each with a narrow brown stripe, apices brown; tibiae brown, each with a pair of spines at ventral apex, mesotibiae with posterior surfaces flat, yellow-brown, glabrous, shiny; tarsi yellow-brown. ***Abdomen***: Five ventrites; ventrite 1 longest, ventrite 4 shortest, ventrites 2, 3, and 5 subequal in length; ventrites 1–3 weakly flattened at midline, ventrites 4 and 5 convex; ventrite 1 with a margined, triangular, intercoxal projection; ventrites 2–4 with lateral margins each produced to form a small, rounded lobe which clasps the epipleuron; ventrites 4 and 5 with moderately deep impressions at anterolateral margins; ventrite 5 apex broadly rounded. Ventrites covered with shallow, closely spaced punctures; ventrite 1 with punctures more widely spaced, ventrites 2–5 with punctures more closely spaced; medial punctures mostly obscured by dense covering of yellow setae, longest at median 1/5 of ventrites 3–5. ***Aedeagus***: Phallobase much shorter than parameres, penis slightly longer than parameres (Fig. 35). Parameres, in dorsal view (Fig. 35A), widest basally; lateral margins weakly arcuate at basal 2/3, then parallel at apical 1/3; median margins straight and moderately divergent at basal 1/2, then arcuate to abruptly narrowed, strongly produced tips at apical 1/3; apices narrow, acute. Penis evenly convergent at basal 3/4, then abruptly narrowed at apical 1/4, apex very narrowly rounded; no visible corona; basal apophyses moderately long, 1/2 as long as phallobase, straight, very broad, blunt at tips. In lateral view (Fig. 35B), penis bent and abruptly angled above parameres near midpoint. Fibula absent.

##### Etymology.

The specific epithet *carinatus*, an adjective in the nominative singular derived from the Latin meaning keeled, refers to the presence of a pair of basal, sublateral carinae on the pronotum.

##### Distribution.

North Queensland, Australia. Known only from the type locality on the north slope of Mt. Elliot (Fig. 8).

##### Habitat.

Geoff Monteith, one of the collectors, described the area thus: “Mt. Elliot is a high, isolated, rainforest-capped mountain with a strikingly unique and endemic fauna” (G. Monteith, in litt.). The specimen was collected using a flight intercept trap at 1000 m elevation.

#### Stetholus elongatus

Taxon classificationAnimaliaColeopteraElmidae

Carter & Zeck, 1929

F47771A4-B40A-5A02-B64F-ADC0CBE45502

Figs 9 , 36 , 37

##### Type locality.

Allyn River at Gresford; 32.350°S, 151.750°E; New South Wales, Australia (holotype deposited in the Australian Museum, Sydney).

##### Paratypes examined

**(5).** Gresford / Allyn R., N.S.W. / Oct. 1926 / H. J. Carter // PARATYPE [blue label] (4 ANIC, 1 SAMA).

##### Other material examined

**(56). Australian Capital Territory.** Kambah Pool / Murrumbidgee / River ACT / 1.i.1978 / J.F.Lawrence (3 ANIC); AUSTRALIA: ACT / Murrumbidgee River / Point Hut Xing S Canberra / 35°33'55"S,149°03'56"E / 1-I-2001, coll. C. B. Barr (9 EMEC, 3 QM); AUSTRALIA: ACT / Murrumbidgee River at / Casuarina, E Cotter Dam / 35°19'41"S,148°57'01"E / 2-I-2001, coll. C. B. Barr (4 ANIC, 9 EMEC, 3 QM); AUSTRALIA: ACT / Gigerbine [Gigerline] NR, Angle / Crossing 31 XII 2000 / Murrumbidgee River (WDS-A-1337 on reverse)// William D. / Shepard, leg. (3 EMEC). **New South Wales.** AUSTRALIA: NSW / Deua NP, Deua River at / Deua River Campground / 35°45'00"S, 149°54'53"E / 4-I-2001, coll. C. B. Barr (8 EMEC); AUSTRALIA: NSW / Deua NP, Deua R / Cmpgd. 4 I 2001 / Deua River (WDS-A-1348 on reverse) // William D. / Shepard, leg. (3 EMEC); Tallong / N.S.W. / FHTaylor (2 ANIC, 4 SAMA); same locality // On submerged / sticks in the / Shoalhaven R. (2 ANIC); AUSTRALIA: NSW / NW of Braidwood / 3 I 2001 / Shoalhaven River (WDS-A-1344 on reverse) // William D. / Shepard, leg. (2 EMEC); Pierce’s Pass, / Blue Mtns., N.S.W. / 5.xii.1971 / G.B.Monteith (1 QM).

##### Differential diagnosis

**(n = 61).***Stetholuselongatus* (Figs 36, 37) can be distinguished from other species of *Stetholus* (Figs 34–35, 38–42) by a combination of the following characters: Antennae distinctly clavate; pronotum moderately sculptured, lacking basal sublateral carinae; metatibia usually with a narrow, elongate bare area of variable length at the posterobasal 1/3; male genitalia (Fig. 37) stout and heavily sclerotized. *Stetholusworonora* (Fig. 41) most closely resembles *S.elongatus* but has short, basal sublateral pronotal carinae. Although the male genitalia of the two species are similarly stout and heavily sclerotized, the penis of *S.elongatus* (Fig. 37) is narrow and tapered at the apex, while that of *S.woronora* (Fig. 42) is wide and bulbous near the apex. *Stetholuslongipennis* (Figs 38) is usually shorter than *S.elongatus* (Fig. 36) (4.1–4.6 mm long vs. 4.7–5.3 mm, excluding the head), has slender, elongate antennae, and the male genitalia are strikingly different (Figs 39, 37).

##### Variation.

The examined females exhibit minor secondary sexual dimorphism with the lateral margin of the elytra slightly explanate at the posterior 1/4 laterad to stria 11; in males, stria 11 is just inside the lateral margin, which is not explanate. The metatibia of both sexes has a posterior, linear bare patch which varies in length but is restricted to the basal 1/2, and nearly always the basal 1/3. This character is occasionally obscure, and is probably the result of abrasion of the setae. Otherwise, except for minor differences in the depth and extent of the pronotal impressions, the specimens examined are quite uniform. Measured specimens vary in size from 4.7–5.3 mm long and 1.8–2.1 mm wide (n = 12). The males and the females are of similar size: males 5.0–5.1 mm long, 1.8–2.1 mm wide (n = 7); females 4.7–5.3 mm long, 1.8–2.1 mm wide (n = 5). Carter & Zeck (1929) reported a body length of 5.0–6.0 mm in their species description which likely included the length of the head.

##### Distribution.

*Stetholuselongatus* occurs in the Australian Capital Territory, New South Wales, and Victoria (A.Glaister, in litt.), Australia (Fig. 9).

##### Habitat and behavior.

The habitat and behavior of this species is as described for the genus. Populations can be enormous in suitable habitats. Specimens also have been collected in light traps (A.Glaister, in litt.).

##### Associated byrrhoid taxa.

Elmidae: Larainae: *Hydoralaticeps*, *Ovolaraaustralis*; Elminae: *Austrolimnius* spp., *Coxelmisnovemnotata*, *Kingolusmetallicus*, *K.tinctus*, *K.* spp., *Notriolusmaculatus*, *N.minor*, *N.setosus*, *N.* spp., *Simsonia* spp. Psephenidae: *Sclerocyphonbasicollis*, *S.minimus*, *S.striatus*.

##### Comments.

As noted in the *Hydoralaticeps* Comments, there are specimens of *S.elongatus* in the >AM, NMV and SAMA which bear locality labels identical to those of *H.laticeps*. Carter & Zeck (1929, 1932) made no mention of the *S.elongatus* specimens from Tallong, or that the two species co-occur. The larva of this species has been reared to the adult by Glaister (A.Glaister, in litt.).

#### Stetholus longipennis sp. nov.

Taxon classificationAnimaliaColeopteraElmidae

2672ACF7-B63C-5F11-A342-AD0AEDC2B95A

http://zoobank.org/4A0A3280-EACD-42E2-9851-7BB81665144D

Figs 10 , 27 , 38 , 39

##### Type locality.

Hunters Creek north of Mount Molloy; 16.6324° S, 145. 3254° E; north Queensland, Australia.

##### Type material.

***Holotype* male.** “AUSTRALIA: Queensland / 5 km N Mount Molloy / 17 I 2001 / Hunters Creek / S16°38’00” E145°19’27” (WDS-A-1368 on reverse) // William D. / Shepard, leg. // HOLOTYPE / Stetholus / longipennis / Barr & Shepard” [red label, handwritten]. Dry pinned. Deposited in the Australian National Insect Collection, Canberra; ANIC Database Number 25-077642. ***Paratypes* (108).** Same data as for holotype (1 >AM, 1 ANIC, 13 EMEC, 1 QM); AUSTRALIA: no. QLD / Hunters Creek at Hwy. 44 / 5 rd. km. N Mount Molloy / 16°38’00”S, 145°19’27”E / 17-I-2001, coll. C. B. Barr (5 EMEC, 1 QM); AUSTRALIA: no. QLD / Bushy Creek at Hwy. 44 / just W of Julatten / 16°36’40”S, 45°20’10”E / 17-I-2001, coll. C.B.Barr (1 EMEC); AUSTRALIA: no. QLD / Emerald Creek at Hwy. 1 / E of Mareeba / 16°59’12”S, 145°28’21”E / 17-I-2001, coll. C.B. Barr (6 EMEC); AUSTRALIA: Queensland / Emerald Creek Store / 17 I 2001 / Emerald Creek / S16°59'12" E145°28'21" (WDS-A-1369 on reverse) // William D. / Shepard, leg. (1 >AM, 9 EMEC); AUSTRALIA: no. QLD / Freshwater, Freshwater / Cr. at Ryan Weare Park / 16°53’13”S, 145°42’05”E / 18-I-2001, coll. C.B. Barr (6 EMEC); AUSTRALIA: Queensland / Freshwater / 18 I 2001 / Freshwater Creek (WDS-A-1370 on reverse) // William D. / Shepard, leg. (2 EMEC); AUSTRALIA: no. QLD / Mulgrave River at Hwy. 1 / 1 rd. km. S of Gordonvale / 17°06’10”S, 145°47’15”E / 18-I-2001, coll. C. B. Barr (5 EMEC, 1 QM); AUSTRALIA: Queensland / 1 km S Gordonvale, 18 I 2001 94 ft / Mulgrave River / (WDS-A-1371 on reverse) // William D. / Shepard, leg. (1 >AM, 1 ANIC, 2 EMEC, 1 QM); AUSTRALIA: no. QLD / Fishery Creek at / Hwy. 1, Fishery Falls / 17°11’10”S, 145°53’11”E / 18-I-2001, C. B. Barr (6 EMEC); AUSTRALIA: Queensland / Fishery Falls / 18 I 2001 / Fishery Creek (WDS-A-1372 on reverse) // William D. / Shepard, leg. (1 >AM, 1 ANIC, 2 EMEC); QLD. Babinda / Apr. 1946 / J.G.Brooks (3 ANIC); 16.03S to 16.05S / 145.28E Cape / Tribulation area / QLD 21-28Mar.1984 / A.Calder & T.Weir // on rocks / in stream (1 ANIC); 16.03S to 16.05S / 145.28E QLD, Cape / Tribulation area / 1-11 May 1992 / J.F.Lawrence // on rocks / in stream (1 ANIC); same data as for preceding // Genitalia prep. / HS-243 ♂ / A.Calder 1997 (1 ANIC); same data as for preceding // Genitalia prep. / HS-274 ♀ / A.Calder 1997 (1 ANIC); QLD. Cardstone / 23 Jan. 1965 / J.G.Brooks / at light (4 ANIC); same data as for preceding // Genitalia prep. / HS-307 ♀ / A.Calder 1999 (1 ANIC); Cardstone, N.Q. / 23.i.65. J.G. & / J.A.G.Brooks // Genitalia prep. / HS-241 ♂ / A.Calder 1997 (1 ANIC); Crystal Cascades / Via Cairns, N.Qld. / 22.xii.1964. / G. Monteith // EX UQIC / DONATED / 2011 (7 QM); same locality / H.A.Rose. (1 QM); Crystal Cascades / Cairns, N.Qld. / 30.xii.1963. / G. Monteith // EX UQIC / DONATED / 2011 (1 QM); QLD. Gordonvale / Apr. 1946 / J.G.Brooks // J. G. Brooks / Bequest, 1976 (4 ANIC); Little Mulgrave R. / Gordonvale, N.Q. / Apr. 1946 / J. G. Brooks // Australian Museum / K 579979 (2 >AM); Mossman, Q. / 25 Mar 1967 / M.S. Upton (1 ANIC); Mossman Gorge / Via Mossman, N.Qld. / 25-26.xii.1964. / G. Monteith // EX UQIC / DONATED / 2011 (2 QM); Mt. Molloy, Q. / Station Creek, at light / 30.xii.69 J.G.Brooks (1 ANIC); 32km S [N?] of Ravenshoe, Q. / (17.38S, 145.29E) / K.Hyde // 12.ii.1966, Genitalia prep. / HS-239 ♂ / A.Calder 1997 (1 ANIC); same data as for preceding // Genitalia prep. / HS-240 ♂ / A.Calder 1997 (1 ANIC); Upper Daintree R. / Via Daintree, / 27.xii.1964. N.Qld. / G. Monteith // EX UQIC / DONATED / 2011 (3 QM); Upper Finch Hatton / Ck., Via Finch / Hatton, N.Qld. / 3.i.1965. / G. Monteith // EX UQIC / DONATED / 2011 (1 QM); Upper Mulgrave River, / 30.iv.1970, N.Qld. / G. B. Monteith // EX UQIC / DONATED / 2011 (2 QM). Paratypes all with the following label: PARATYPE / *Stetholus* / *longipennis* / Barr & Shepard [yellow label, printed].

##### Other material examined

**(17).** Archers Ck., Q. / Mt. Garnet Rd., / 28.xii.1964 / J.G.Brooks (1 ANIC); Bellenden Ker Range, NQ / Cableway Base Stn, 100m / 17 Oct.-9 Nov. 1981 / EARTHWATCH/QLD. MUSEUM / MV light, rainforest // A.N.I.C. / COLEOPTERA / Voucher No. / 83-0610 [green label] // Stetholus / sp. 1 / det. T. A. Weir 1983 (1 QM); Cardstone, N.Q. / 23.i.65. J.G. & / J.A.G.Brooks (4 ANIC); Cardstone QLD / 10-13.iii.1966 / K. Hyde (1 ANIC); Henrietta Ck., / Palmerston Nat. / Pk., N.Qld. / 29.xii.1964. / G. Monteith // EX UQIC / DONATED / 2011 (1 QM); Millstream at Archers / Ck. N. Q. Mt. Garnet / Rd. 28.xii.64 / J.G. Brooks (1 ANIC); same data as for preceding // Genitalia prep. / HS-306 ♀ / A.Calder 1999 (1 ANIC); same data as for preceding // Genitalia prep. / HS-308 ♀ / A.Calder 1999 (1 ANIC); Mossman Gorge / Via Mossman, N.Qld. / 25-26.xii.1964. / G. Monteith // EX UQIC / DONATED / 2011 (1 QM); 3 mls.W. of / Mossman, Q. / 14 Mar. 1964 / I.F.B.Common / & M.S.Upton (1 ANIC); 32km S [N?] of Ravenshoe, Q. / (17.38S, 145.29E) / K.Hyde // 16.ii.1966 (3 ANIC); Spring Ck. N. Q. / Heberton Rd. / 30.xii.64 / J.G. Brooks (1 ANIC).

##### Differential diagnosis.

*Stetholuslongipennis* (Fig. 38, 39) can be distinguished from other species of *Stetholus* (Figs 34–37, 40–42) by a combination of the following characters: Length usually shorter than or equal to 4.6 mm; antennae slender, almost moniliform; pronotum smooth or lightly sculptured, sublateral carinae absent; metatibiae entirely setose; male genitalia with penis very slender and much longer than parameres. Conversely, *S.metatibialis* (Fig. 40) and *S.carinatus* (Fig. 34) both have long, distinct sublateral pronotal carinae; *S.woronora* (Fig. 41) has very short carinae; and all three have distinctly clavate antennae. *Stetholuselongatus* (Fig. 36) also lacks pronotal carinae, but is usually longer than 4.6 mm and the antennae are clavate. The male genitalia of *S.longipennis* (Fig. 39) are strikingly different from those of other *Stetholus* (Figs 35, 37, 42) excluding *S.metatibialis* for which males are currently unknown.

##### Description

**(n = 127). *Body***: Size 4.1–4.6 long, 1.6–1.8 wide (n = 21). Color dark brown to black dorsally and ventrally, but appearing lighter due to layer of dense, short, yellow setae; first two antennomeres, trochanters, basal 2/3–3/4 of femora yellow; apical antennomeres, coxae, tibiae, tarsi dark brown. ***Head***: Punctures shallow, fine, evenly spaced; setae fine, yellow, recumbent to erect. Vertex with faint V- or U-shaped impression, open anteriorly, extending from near antennal bases to a distinct median impression; frontoclypeal suture arcuate. Antenna with eleven antennomeres; antennomere 1 longest, 3 × longer than wide, curved; antennomere 2 spherical; both with long, curved setae; antennomere 3 ovoid; antennomeres 4–11 each subspherical, widening slightly towards antennal apex, forming a slender, elongate, almost moniliform, club. Eye finely-faceted, suboval at base, moderately protuberant; dorsal and posteroventral margin with fringe of long, curved, black setae. Clypeus convex, broadly rectangular, emarginate anteriorly, densely setose. Labrum rectangular, longer and slightly narrower than clypeus; setose; anterior margin weakly emarginate, with band of short, yellow setae; lateral margins each with a wide, dense band of long, curved setae. Mandible with two teeth, outermost acute apically, innermost truncate apically; prostheca shelf-like, very thin, apically with coarse, long setae; molar area large, moderately oval, surface striate; lateral edge basally with a partially free lobe with several thin setae. Maxillary palpus black, setose, with four palpomeres; palpomere 1 short, annular, with inner apical border spine-like; palpomere 2 twice as long as wide; palpomere 3 as long as 2, wider apically; palpomere 4 wide, ventral surface with a subcircular, concave, white sensory area angled obliquely from the apex to the base. Labial palpus black, glabrous, with three palpomeres; palpomeres 1 and 2 short, annular; palpomere 3 longer, wider, quadrate, apex truncate with an oval, concave, white sensory area. ***Pronotum***: Shape generally trapezoidal, wider than long, widest at base; length 0.8–1.0 mm, width 1.2–1.3 mm; disc with distinct punctures evenly spaced ~ 1 diameter apart, deeper near lateral margins; densely setose, with short, fine, yellow setae and longer, coarser, dark setae. Anterior margin arcuate; anterior angles obsolete; lateral margins bisinuate; posterior angles 90°, blunt, depressed; posterior margin weakly trisinuate. Disc slightly convex; shallow, broadly V-shaped, transverse impression at apical 1/4–1/2; basal 1/2 with two faint to distinct, oblique, lateral depressions and two large, deep prescutellar foveae; pronotal margin inflated posterior to fovea. ***Scutellar shield***: Cordate; posterior apex slightly raised; finely setose. ***Elytron***: 3.3–3.6 mm long, 0.8–0.9 mm wide. Elytra conjointly ~ 2 × as long as wide, widest near posterior 1/3; apices together forming a triangular notch between. Humerus prominently inflated, elytral base depressed medially; disc at 1/4–1/3 distance from base with a shallow depression from suture to stria 5. Disc with 10 punctate, weakly impressed striae, intervals flat; punctures very fine, spaced one diameter apart, obsolete apically; accessory basal stria between striae 1 and 2 long; stria 3 ending before apex; striae 4 and 5 joining before apex; striae 10 and 11 joining and ending before apex. ***Metathoracic wings***: Macropterous. ***Prosternum***: Very short anterior to procoxae; prosternal process moderately narrow, 3 × longer than wide, margined, with a median longitudinal carina, apex narrowly rounded. ***Mesoventrite***: Short; disc convex between mesocoxae with a deep mesoventral cavity to receive prosternal process. ***Metaventrite***: Broadly rectangular; disc with a median, round concavity near anterior margin and a wide median depression covering posterior 1/2–2/3; discrimen extending from concavity to posterior margin; metakatepisternal suture distinct; disc with small, shallow, variably spaced punctures mostly obscured by dense, fine, recumbent, yellow setae. ***Legs***: Of similar lengths; each leg with femur and tibia subequal in length; tarsus slender, with tarsomere 5 slightly longer than tarsomeres 1–4 combined; claws simple, moderately large, sharply acute. Coxae dark brown, metacoxae deeply sulcate; femora yellow, dorsal surfaces of each with a narrow brown stripe, apical 1/4–1/3 dark brown; tibiae dark brown, mesotibiae with posterior surfaces nearly glabrous, shiny; metatibiae entirely setose, weakly arcuate. ***Abdomen***: Five convex ventrites, each with a shallow depression near lateral margin; ventrite 1 with a margined, broadly triangular, intercoxal projection; ventrites 2–4 broadly rectangular, with lateral margins each produced to form a small lobe of varying size, largest on ventrites 3 and 4, which clasps the epipleuron; posterior border of ventrite 5 with a broadly rounded apex. Ventrites covered with shallow punctures spaced 1–2 diameters apart, mostly obscured by dense covering of yellow setae; setae longer at median 1/5 of ventrites 3–5. ***Aedeagus***: Phallobase lightly sclerotized, open dorsally, fused to short parameres; penis very long, nearly as long as phallobase and parameres together (Fig. 39). In dorsal view (Fig. 39A), parameres with lateral margins sinuate, convergent from junction with phallobase to near apex then strongly divergent and curved laterally; median margins nearly parallel at basal 2/3 then strongly divergent; apices acute. Penis beyond paramere tips with lateral margins nearly parallel almost to acute apex; no visible corona; dorsal median longitudinal carina with darker sclerotization present; basal apophyses very long, nearly as long as phallobase, straight, very broad, blunt at tips. In lateral view (Fig. 39B), paramere triangular, moderately convex dorsally, nearly flat ventrally; apex broadly rounded to truncate with a few, small, irregular teeth; penis curved, tip ventrally directed. Fibula absent. **Ovipositor**: Moderately sclerotized; oval in outline, 2 × longer than wide; baculum ~ 2 × longer than gonocoxite; proximal gonocoxite subrectangular, wide and short; distal gonocoxite narrow and short, ~ 2 × as long as wide, length equal to proximal gonocoxite length, median and lateral margins arcuate, together separate basally but contiguous medially to apices; stylus very narrow.

##### Variation.

The females exhibit minor secondary sexual dimorphism with the lateral elytral margin slightly explanate at the posterior 1/4 laterad of stria 11; in males, stria 11 is just inside the lateral margin, which is not explanate. Measured specimens vary from 4.1–4.6 long and 1.6–1.8 wide (n = 21). The females are slightly larger than the males: females 4.3–4.6 mm long, 1.8 mm wide (n = 8); males 4.1–4.5 mm long, 1.6–1.8 mm wide (n = 13). The width of the prosternal process varies a bit between individuals. Otherwise, except for minor differences in the depth and extent of pronotal impressions, the specimens are quite uniform.

##### Etymology.

The specific epithet *longipennis*, an adjective in the nominative singular derived from the Latin *longi* (long) plus *pennis* (penis), refers to the male genitalia in which the length of the penis greatly exceeds the length of the parameres (Fig. 39).

##### Distribution.

*Stetholuslongipennis* occurs in north and central Queensland, Australia (Fig. 10).

##### Habitat and behavior.

*Stetholuslongipennis* was collected by the authors from medium to large sand-bottomed streams with logs and debris, some with boulders, and a small river at elevations ranging from 5–417 m (Fig. 27). All had clear water which varied from warm to cool. The beetles were collected from logs and rocks in fast current or rapids, and from a spillway. The adults fly readily from the net, and also have been taken at lights. At the type locality, Hunters Creek, the stream was well-shaded, with many logs and much debris, and many *S.longipennis* were concentrated on a concrete spillway below a bridge.

##### Associated byrrhoid taxa.

Elmidae: Larainae: *Australaraglaisteri* sp. nov., *Ovolaralawrencei* sp. nov., *O.leai*, *O.monteithi* sp. nov., *Potamophilinuspapuanus*; Elminae: *Austrolimnius* spp., *Graphelmispallidipes*, *Kingolus* spp., *Notriolustaylori*, *Notriolus* spp., *Simsonia* sp. Psephenidae: *Sclerocyphonbasicollis*, *Sclerocyphonminimus*.

#### Stetholus metatibialis sp. nov.

Taxon classificationAnimaliaColeopteraElmidae

CC56EB3A-8284-5A8E-9F92-BE15DDF1B94D

http://zoobank.org/37506188-9496-40B4-BC38-10BE15FF63D3

Figs 11 , 40

##### Type locality.

Mt. Bellenden Ker northwest of Babinda; 17.2672° S, 145.8700° E; Wooroonooran National Park, north Queensland, Australia.

##### Type material.

***Holotype* female.** “Bellenden Ker Range, NQ / Cable Tower 3 [now Tower 6], 1054m / 17 Oct.-5 Nov. 1981 / EARTHWATCH/QLD. MUSEUM // A.N.I.C. / COLEOPTERA / Voucher No. / 83-0611” [green label] // “HOLOTYPE / Stetholus / metatibialis / Barr & Shepard” [red label, handwritten]. Dry pinned. Deposited in the Queensland Museum, South Brisbane; Registration Number QM T250615.

##### Differential diagnosis.

The single female specimen of *S.metatibialis* (Fig. 40) is characterized by an elongate-oval body shape; labrum “moustache” composed of two discrete, lateral tufts of very long, dark, curved setae (setal origin unclear, possibly mandibular) (Fig. 40C); long pronotal basal sublateral carinae; and posterior surfaces of both the meso- and metatibiae glabrous and shiny. While other species of *Stetholus* may have similar labral tufts, none are as long and distinctive. The metatibia of *S.elongatus* (Fig. 36) has a narrow, elongate, posterior bare area of variable length, usually at the basal 1/3, as opposed to that of *S.metatibialis* in which the posterior surface is entirely bare; the metatibiae of the others are entirely setose. *Stetholuselongatus* and *S.longipennis* (Fig. 38) lack pronotal sublateral carinae. *Stetholusmetatibialis* (Fig. 40) bears a superficial resemblance to *Ovolara* species (Figs 23–26, 28–31) because of its elongate-oval body and strongly punctate elytra, however it is easily separated by the very short prosternum anterior to the procoxae (vs. prosternum long, extended anteriorly) and the presence of a transverse pronotal impression (vs. no impression).

##### Description

**(n = 1). Holotype female. *Body***: Size 3.9 mm long, 1.4 mm wide; elongate-oval. Dorsal color dark brown; head black; venter mostly brown; first two antennomeres, posterior metaventrite, coxae, trochanters, femora, posterior face of meso- and metatibiae yellow-brown. Setae of dorsal surfaces short, yellow, semi-erect and recumbent, setae of ventral surfaces long and recumbent. ***Head***: Densely setose and punctate, punctures < 1 diameter apart or nearly contiguous. Vertex with a faint V-shaped impression, open anteriorly, extending from antennal bases towards occiput; frontoclypeal suture straight, obscure. Antenna with 11 tomentose antennomeres; antennomeres 1 and 2 yellow-brown with coarse, yellow setae; antennomere 1 longest, ~ 3 × longer than wide, curved; antennomere 2 ovoid; antennomeres 3–11 brown with dense yellow setae, together forming a tight, elongate club; antennomeres 7–11 of equal width, antennomere 11 longer than all but antennomeres 1 and 2, apex bluntly rounded. Eye finely faceted, suboval at base, weakly protuberant; dorsal margin with fringe of long, curved setae. Clypeus transverse, convex, anterior margin straight; disc densely setose, lateral margins with longer setae. Labrum trapezoidal, wider than long, 2 × longer and slightly narrower than clypeus; densely setose; anterior margin weakly emarginate with band of short, yellow setae; lateral margins with dense fringes of long, yellow setae, each margin with a discrete tuft of very long, dark, curved setae extending to maxilla (setal origin unclear, possibly mandibular). Maxillary palpus with four setose palpomeres; palpomere 1 yellow, short, annular; palpomere 2 yellow, 2 × as long as wide; palpomere 3 yellow, nearly as long as 2, wider apically; palpomere 4 brown, longest and widest, ovoid, ventral surface with a broadly oval, slightly concave, pale sensory area angled obliquely from the apex to the base. Labial palpus yellow, glabrous, with three palpomeres; palpomeres 1 and 2 yellow, annular, short and narrow; palpomere 3 brown, conical, much longer and wider than others, apex truncate with a narrowly oval, flat, slightly concave, pale sensory area. *P****ronotum***: Shape generally trapezoidal, slightly wider than long, widest at base; 1.0 mm long, 1.1 mm wide; disc densely punctate, punctures evenly spaced ~ 1 diameter apart. Anterior margin arcuate; anterior angles obsolete; lateral margins sinuate and arcuate, moderately explanate; posterior angles raised, protruding, acute, posterior margin weakly trisinuate. Disc weakly convex, more convex at basal 1/2; distinct, transverse V-shaped impression at apical 1/3–1/2; two distinct, basal, sublateral carinae 1/3–1/2 as long as pronotum, bordered by shallow medial impressions and lateral excavations; two shallow, indistinct prescutellar foveae. ***Scutellar shield***: Cordate, longer than wide, apex rounded; flat; densely setose. ***Elytron***: 2.9 mm long, 0.7 mm wide. Elytra conjointly 2 × as long as wide; anterior 2/3 almost parallel-sided; margins narrowly marginate. Humerus inflated, elytral base depressed medially; disc convex at anterior 1/3, flattened at 1/3–1/2 distance from base, then weakly convex to apex. Disc with ten strongly punctate, weakly impressed striae, intervals slightly raised, sutural interval more so; accessory basal stria of 6 punctures between striae 1 and 2 short; striae 3 and 4 join near apex; disc punctures large and deep at basal 2/3, becoming much smaller and shallower towards apex, separated by one diameter. ***Prosternum***: Very short anterior to procoxae, marginate anteriorly. Prosternal process moderately narrow, long, 4 × longer than wide; nearly parallel-sided between coxae then slightly widened towards rounded apex; laterally marginate, medially sulcate at basal 1/2, carinate at apical 1/2; surface tomentose. ***Mesoventrite***: Short, marginate, densely setose, with a deep mesoventral cavity to receive prosternal process. ***Metaventrite***: Broadly rectangular; very setose; anterior margin marginate, bordered posteriorly by a small, transverse excavation; disc with discrimen extending almost from anterior to posterior margin, deeply incised at posterior 2/3; disc laterad to discrimen very convex; metakatepisternal suture distinct. Disc laterally with large, variably spaced punctures; punctures obscured medially by a broad, triangular patch of very long, dense, recumbent, yellow-orange setae. ***Legs***: Of similar lengths; each leg with femur and tibia nearly subequal in length; foreleg stouter than the others; tarsus with tarsomere 5 longer than tarsomeres 1–4 combined, distinctly expanded at 1/3 distance to apex; claws simple, large, sharply acute. Pro- and mesocoxae yellow; metacoxae yellow medially, brown laterally, deeply sulcate; femora yellow, dorsal surfaces of each with a narrow brown stripe, apices brown; tibiae brown, meso- and metatibiae with posterior surfaces yellow-brown, glabrous, shiny; tarsi brown. ***Abdomen***: Five ventrites; ventrites 2 and 3 subequal in length, ventrite 4 shortest, ventrite 5 longest; ventrites convex; ventrite 1 with a wide, triangular, intercoxal projection; ventrites 2–4 with lateral margins each produced to form a small, rounded lobe which clasps the epipleuron; ventrites 3 and 4 depressed basally, raised at posterior margins; ventrite 5 with impressions at basomedial and basolateral margins, apex rounded. Ventrites covered with shallow punctures variably spaced one or more diameters apart; punctures of ventrites 3–5 medially obscured by dense covering of yellow setae.

##### Etymology.

The specific epithet *metatibialis* is an adjective in the nominative singular derived from the Greek *meta* meaning after or posterior, and the Latin *tibia*, the lower portion of a leg. *Metatibialis* points to the diagnostic character present on the hind tibia, specifically, the glabrous posterior surface (Figs 40D).

##### Distribution.

North Queensland, Australia. Known only from the type locality in the Bellenden Ker Range in Wooroonooran National Park, west of Bellenden Ker and northwest of Babinda (Fig. 11).

##### Habitat.

The single specimen was taken at UV light trap at an elevation of 1054 m on the east slope of Mt. Bellenden Ker. According to the project leader “the whole place is solid rainforest and there are many endemics at higher elevations” (G. Monteith, in litt.).

#### Stetholus woronora sp. nov.

Taxon classificationAnimaliaColeopteraElmidae

82FEB6C7-A2A1-56BB-8D63-7ADBF068E05A

http://zoobank.org/8185A9F0-AE2F-4945-B5AF-AA78CB6F55BD

**Figure 43.**
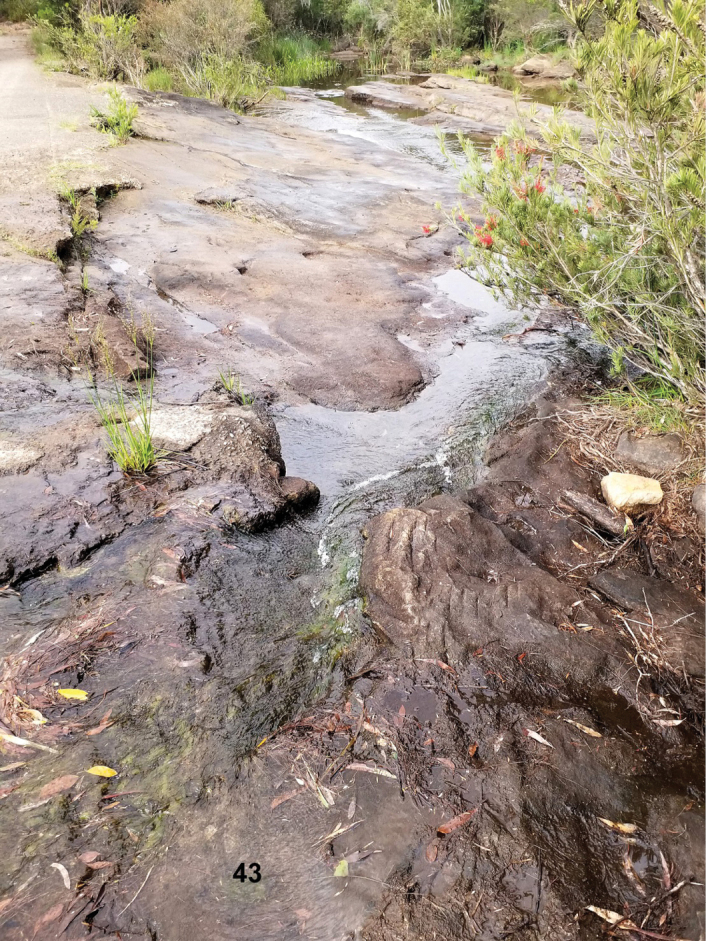
*Stetholusworonora* sp. nov., type locality: Woronora River, north of Engadine, NSW, Australia (photograph courtesy of Martin Fikáček, National Museum, Prague, Czech Republic).

Figs 12 , 41–42 , 43

##### Type locality.

Woronora River north of Engadine; 34.0465° S, 151.0062° E; New South Wales, Australia (Fig. 43).

##### Type material.

***Holotype* male.** “AUSTRALIA: NSW / Woronora River, N / Engadine, S Sydney / -34.04652, 151.00621 / 10 m, 23.xi.2019 // Fikáček, Seidel / & Sýkora lgt. / AU-2019-34 // HOLOTYPE / Stetholus / woronora/ Barr & Shepard” [red label, handwritten]. Dry pinned. Deposited in the Australian National Insect Collection, Canberra; ANIC Database Number 25-077643. ***Paratypes* (9).** Same data as for holotype (4 ANIC, 3 EMEC, 2 NMPC). Paratypes all with the following label: PARATYPE / *Stetholus* / *woronora* / Barr & Shepard [yellow label, printed].

##### Differential diagnosis.

*Stetholusworonora* (Figs 41, 42) can be distinguished from other species of *Stetholus* (Figs 34–40) by a combination of the following characters: Body large, > 5.0 mm long; antennae clavate; pronotum with very short, basal sublateral carinae; male genitalia stout and heavily sclerotized. *Stetholuselongatus* (Fig. 36) most closely resembles *S.woronora* but lacks pronotal carinae; the male genitalia are somewhat similar but the penis of *S.elongatus* (Fig. 37) is narrow and tapered at the apex whereas that of *S.woronora* (Fig. 42) is wide and bulbous. The other three species of *Stetholus* are much shorter (4.5 mm or less) and do not have similarly stout, heavily sclerotized genitalia (those of *S.metatibialis* are unknown). Furthermore, *S.longipennis* (Fig. 38) lacks sublateral pronotal carinae; *S.metatibialis* (Fig. 40) and *S.carinatus* (Fig. 34) both have much longer, more prominent carinae.

##### Description

**(n = 10). *Body***: Size 5.2–5.6 mm long, 1.9–2.2 mm wide (n = 9). Color black except first two antennomeres, trochanters and basal 2/3 of femora yellow-brown or light red-brown. All surfaces covered with short pale yellow or longer black setae. ***Head***: Heavily punctate and setose, with many long, erect and semierect, curved, black setae and much shorter pale yellow setae. Eye finely faceted, suboval at base, weakly protuberant; with a dorsal and posteroventral fringe of long, curved, black setae. Antenna with eleven antennomeres; antennomere 1 3 × longer than wide, curved; antennomere 2 spherical; both with long, curved, black setae; antennomeres 3–11 forming an elongate club. Frons with a distinct Y-shaped impression, upper arms nearly reaching antennal bases, frons depressed between; frontoclypeal suture straight. Clypeus broadly rectangular, emarginate anteriorly, convex, densely setose. Labrum narrower than clypeus, trapezoidal, widest at base, weakly emarginate; anterior margin with band of short, pale yellow setae, lateral margins with long, dense brushes of light and dark, curved setae. Maxillary palpus black, setose, with four palpomeres; palpomere 1 short; palpomeres 2 and 3 longer; palpomere 4 longer and wider, ventral surface with a circular to oval, concave, white sensory area angled obliquely from the apex to the base. Labial palpus black, glabrous, with three palpomeres; terminal palpomere quadrate, apex with oval white sensory area. ***Pronotum***: Shape generally trapezoidal, slightly wider than long, widest at base; 1.1–1.3 mm long, 1.3–1.5 mm wide; disc heavily and evenly punctate and setose; punctures deep, spaced mostly 1 diameter apart; setae either short, fine, pale yellow or longer, stout, dark. Anterior margin arcuate; anterior angles obsolete; lateral margins weakly trisinuate; posterior angles 90°, depressed; posterior margin weakly trisinuate. Disc with a shallow, broadly V-shaped, transverse impression from anterior 1/3–1/2 contiguous with a short, median, longitudinal impression; two shallow, oblique, lateral impressions at posterior 1/3; two very short, basal, sublateral carinae; two deep prescutellar foveae. ***Scutellar shield***: Subtriangular with margins weakly arcuate, apex acute, raised; velvety in appearance due to very dense, short, pale yellow setae unlike that of pronotum and elytra. ***Elytron***: 4.0–4.4 mm long, 1.0–1.1 mm wide. Elytra conjointly 2 × as long as wide, slightly widened at apical 1/3; apices together evenly rounded with a small notch between. Setae short, fine, pale yellow. Humerus prominently swollen; anterior margin narrowly depressed inside of humerus, especially at base of interval 6; disc at 1/3 distance from base with a shallow depression from suture to stria 4. Disc with 10 punctate striae; punctures small and spaced < 1 diameter apart, stronger anteriorly, fainter posteriorly; accessory basal stria between striae 1 and 2 long; stria 3 ending before posterior margin, striae 4 and 5 joining and ending just before posterior margin. ***Metathoracic wings***: Macropterous. ***Prosternum***: Very short anterior to procoxae, margined; prosternal process very setose, moderately narrow, 3 × longer than wide, with a distinct median longitudinal carina, apex rounded. ***Mesoventrite***: Short, wide; disc concave between mesocoxae, with a deep mesoventral cavity to receive prosternal process; covered with short yellow setae. ***Metaventrite***: Broadly rectangular; with a wide, circular, median depression covering posterior 3/4; discrimen as long as median depression; metakatepisternal suture present; disc heavily punctate, punctures small and often contiguous; disc covered with short, dense, yellow setae. ***Legs***: Of different lengths, fore leg shortest, hind leg longest; each leg with femur shorter than tibia; mesotibia narrower than pro- and metatibia; tarsus with tarsomere 5 slightly longer than tarsomeres 1–4 combined; claws large, simple, acute. Coxae black, metacoxae deeply sulcate; femora yellow-brown or light red-brown each with dorsal surfaces and apical 1/3 black; tibiae black; mesotibiae with posterior surfaces flat, glabrous, shiny; metatibiae weakly arcuate; tarsi black. ***Abdomen***: Five convex ventrites, each with a shallow depression near lateral margin; ventrite 1 with margined, broadly triangular, intercoxal projection; ventrites 2–4 widely rectangular, with lateral margins each produced to form a small lobe of varying size, those of ventrites 3 and 4 largest, which clasps the epipleuron; ventrite 5 with lateral margins evenly curved with broadly rounded apex. Ventrites covered with shallow, often contiguous, punctures, and semi-erect and recumbent yellow setae. ***Aedeagus***: Mostly well-sclerotized; short, broad, widest at apex of phallobase; phallobase longer than parameres, parameres slightly shorter than penis; phallobase open dorsally (Fig. 42). In dorsal view (Fig. 42A), parameres broad at base, abruptly digitate at apices; medial margin darkly sclerotized at basal 2/3 due to folding of the margin ventrally, inner surface flat against penis; medial margin weakly divergent at basal 1/2, arcuate and strongly divergent at apical 1/2, sinuate before apex; lateral margins evenly arcuate with four, small, sharp teeth near apex. Penis very broad, less well-sclerotized than parameres, medially inflated at basal 2/3 between parameres forming a longitudinal, margined, flat-topped carina; apex bell-shaped, tip produced and bent ventrally; no visible corona; basal apophyses moderately long, 1/2–2/3 as long as phallobase, straight, very broad, transversely truncate at tips. In lateral view (Fig. 42B), aedeagus widest midway between apex and base; paramere broadly triangular in outline, moderately convex dorsally, moderately concave ventrally, apex acute; penis apex narrowly rounded, curved ventrally slightly above paramere apex. In ventral view (Fig. 42C), parameres with arcuate median margins, thickened at basal 1/3; penis with tip longitudinally carinate, fibula absent. ***Ovipositor***: Well-sclerotized; elongate, 2.5 × longer than wide; baculum 1/4 longer than gonocoxite; proximal gonocoxite subrectangular except widened medially at base, with two teeth; distal gonocoxite 2 × longer than proximal gonocoxite, base 2 × wider than apex, apex broader than middle, median margins straight, lateral margins sinuate; stylus narrow, 1/4–1/3 length of distal gonocoxite.

##### Variation.

Females exhibit minor secondary sexual dimorphism with the lateral margin of the elytra very slightly explanate at the posterior 1/4 laterad to stria 11; in males, stria 11 is just inside the lateral margin, which is not explanate. The specimens vary from 5.2–5.6 mm long and 1.9–2.2 mm wide (n = 9). Males are slightly larger than the females, but the sample size is small, particularly for females: males 5.3–5.6 mm long, 2.0–2.2 mm wide (n = 6); females 5.2–5.5 mm long, 1.9–2.1 mm wide (n = 3). Except for small differences in the depth and extent of the pronotal impressions, the specimens are otherwise quite uniform.

##### Etymology.

The specific epithet *woronora*, a noun in the genitive case, refers to the type locality of the species, the Woronora River (Fig. 43). Woronora is an Aboriginal place name meaning black rocks in the Dharug (or Darug) language.

##### Distribution.

New South Wales, Australia. Known only from the type locality south of Sydney (Fig. 12).

##### Habitat.

Although the Woronora River normally has pools interspersed with riffles at the type locality, collector Sýkora (in litt.) reported that due to a severe drought “the river there is pretty much just a small stream and we were surprised there was still some water, given the drought at that time.” The specimens were obtained by “water collecting in a small rapids of a small stream in rocky pool” (Sýkora, in litt.) at an elevation of 10 m (Fig. 43).

##### Associated byrrhoid taxa.

Elmidae: Elminae: *Kingolus* sp., *Notriolus* sp., *Simsonia* sp.

## Discussion

### Distribution, biogeography, and biodiversity

The family Elmidae has both high species richness and endemicity in Australia, but most of that is found in the subfamily Elminae rather than in the Larainae. The vast majority of the described species of both subfamilies are known from along the eastern coast of the continent from Queensland to Victoria. A few elmine species occur in other states, but laraines are known only from Queensland, New South Wales, the Australian Capital Territory, and Victoria. Although island state Tasmania shares five elmine species with the main continent, laraines are apparently absent. This could be an oversight because Tasmania has many streams and rivers, some most likely with suitable habitat.

The Australian Wet Tropics bioregion, characterized by high seasonal rainfall, extends along the northeast coast of Queensland from Cooktown to near Townsville, and is topographically varied with mountain ranges containing deep gorges and fast-flowing rivers which quickly descend to the coastal plain. Although it encompasses only 0.01 % of Australia, the Wet Tropics sustains a large proportion of the continent’s terrestrial plant and vertebrate species, 25 % of which are regional endemics (McKie et al. 2005). The region was placed on the World Heritage list in 1988 in recognition of the high biodiversity and endemism of its rainforest flora and fauna https://www.environment.gov.au/heritage/places/world/wet-tropics.

Intensive surveys of the insects and other invertebrates of the Wet Tropics conducted in the 1980s focused on species diversity, altitudinal zonation, faunal turnover, and biogeography (Yeates and Monteith, 2008). Numerous studies involving aquatic insects were reviewed by Connolly et al. (2008), who concluded that the biodiversity of Wet Tropics streams is high compared to the rest of the continent. The elmids are no exception to this pattern of high biodiversity. Of the 12 species of Larainae known from Australia, eight are found only in north Queensland, including six of the seven newly described species. Five species of laraines, a surprising number, were identified from two rivers and their tributaries, the Daintree and the Mulgrave. It has been determined that the aquatic invertebrate fauna of the Australian Wet Tropics is chiefly of Gondwanan origin, but it also contains some Asian-derived elements (Connolly et al. 2008, McKie et al. 2005).

The island of New Guinea and Australia are part of the same continental land mass which separated from Gondwana ~ 96 mya. They formed a single, continuous landmass during the Pleistocene ice age ~ 18,000 years ago until rising sea levels separated them ~ 10,000 years ago. Today, only ~ 150 km separate the tip of the Cape York Peninsula in far north Queensland from Papua New Guinea. Therefore, it is not surprising to find shared fauna between the two, including unusual mammal groups (monotremes and marsupials) and several insect taxa (Yeates and Monteith 2008, Surbakti et al. 2021). Nonetheless, phylogenetic studies of Wet Tropics invertebrates suggest that species from New Guinea and far northern Australia have had little influence (Yeates and Monteith 2008). Instead, their closest relatives are found in coastal rainforests further south in Queensland and New South Wales. So far only one species of laraine shared with New Guinea has been found, *Potamophiluspapuanus*. This species has not only been collected from the Wet Tropics, but also from the Iron Ranges further north in the Cape York Peninsula, an area that shows a much greater faunal overlap with New Guinea (Yeates and Monteith 2008). Besides *Potamophilinus*, the elmine genera *Austrolimnius* Carter & Zeck, *Coxelmis* Carter & Zeck, *Graphelmis* Delève, and *Simsonia* Carter & Zeck occur both in Australia and New Guinea. Other laraine genera that occur in New Guinea are *Parapotamophilus* Brown and *Potamophilus* Germar, with one and two species, respectively. It would be interesting to discover if any of these taxa are shared as well.

### Exploring Australian elmid biodiversity: past research and future potential

Taxonomic research on Australian elmids was dominated by H. J. Carter and E. H. Zeck from 1926–1948, who described many new genera and species, including three of the four laraine species. H. E. Hinton named many more species in his monograph on *Austrolimnius*, an elmine, in 1965. Until now, there has been a 50+ year hiatus since any new Australian elmid taxa have been described. In the interim, ground-breaking descriptive work on the larval fauna was undertaken by Alena Glaister who developed techniques for rearing larvae to adults, thus establishing associations and enabling larval identification (Glaister 1985, 1992, 1999).

Australia has experienced very little focused elmid collecting, both historically and currently, and its elmid diversity is not very well known. The early elmid researchers (e.g., H. J. Carter, E. H. Zeck, H. E. Hinton) worked predominately with museum specimens or relied on local naturalists to send them material. In the 1980s, through the efforts of Geoff Monteith and others who extensively surveyed the Wet Tropics insect and invertebrate fauna, many elmids were captured primarily with light traps or flight intercept traps (Yeates and Monteith 2008). Since then, only a few collectors have added elmid specimens to museum collections. Aquatic invertebrates, particularly the Ephemeroptera, Trichoptera, and Chironomidae (Diptera), have been the focus of numerous surveys as well as ecological studies in the past (Connolly et al. 2008, McKie et al. 2005), but elmids have gotten at most passing mention in the literature despite their abundance in suitable habitats. An exception is a paper on the role of a few species in wood decomposition (McKie and Cranston 1998).

The results of our limited fieldwork in Australia speak volumes to the opportunity for future survey work and taxonomic research, as our experience with laraines in the Wet Tropics of north Queensland illustrates: During a two day period, at seven stream/river sites on major roads, we collected an undescribed genus, three undescribed species, and a species not yet reported from Australia. Three more new species from that region were found in loan material from the Queensland Museum. Likewise, our elmine collections from north Queensland have proven mostly impossible to identify, and undoubtedly contain many undescribed species because the old taxonomic literature contains relatively few species from north Queensland. Even the more thoroughly explored states of New South Wales and Victoria still hold surprises: *Hydoralaticeps*, previously known only from the type locality for nearly 90 years, was identified from museum specimens collected near Canberra and in coastal Victoria. And most unexpected of all, *Stetholusworonora* sp. nov. was found just two years ago in the suburbs of Sydney.

## Supplementary Material

XML Treatment for Australara

XML Treatment for Australara glaisteri

XML Treatment for Hydora

XML Treatment for Hydora laticeps

XML Treatment for Ovolara

XML Treatment for Ovolara australis

XML Treatment for Ovolara lawrencei

XML Treatment for Ovolara leai

XML Treatment for Ovolara monteithi

XML Treatment for Potamophilinus

XML Treatment for Potamophilinus papuanus

XML Treatment for Stetholus

XML Treatment for Stetholus carinatus

XML Treatment for Stetholus elongatus

XML Treatment for Stetholus longipennis

XML Treatment for Stetholus metatibialis

XML Treatment for Stetholus woronora
